# Pathway and Length Control of Supramolecular Polymers in Aqueous Media via a Hydrogen Bonding Lock

**DOI:** 10.1002/anie.202012710

**Published:** 2020-12-22

**Authors:** Ingo Helmers, Goutam Ghosh, Rodrigo Q. Albuquerque, Gustavo Fernández

**Affiliations:** ^1^ Organisch-Chemisches-Institut Westfälische-Wilhelms-Universität Münster Correnstrasse 40 48149 Münster Germany; ^2^ Lehrstuhl für Systemverfahrenstechnik Technical University of Munich (TUM) Gregor-Mendel-Strasse 4 85354 Freising Germany

**Keywords:** amphiphilic systems, aqueous self-assembly, BODIPY dyes, controlled supramolecular polymerization, hydrogen bonding

## Abstract

Programming the organization of π‐conjugated systems into nanostructures of defined dimensions is a requirement for the preparation of functional materials. Herein, we have achieved high‐precision control over the self‐assembly pathways and fiber length of an amphiphilic BODIPY dye in aqueous media by exploiting a programmable hydrogen bonding lock. The presence of a (2‐hydroxyethyl)amide group in the target BODIPY enables different types of intra‐ vs. intermolecular hydrogen bonding, leading to a competition between kinetically controlled discoidal H‐type aggregates and thermodynamically controlled 1D J‐type fibers in water. The high stability of the kinetic state, which is dominated by the hydrophobic effect, is reflected in the slow transformation to the thermodynamic product (several weeks at room temperature). However, this lag time can be suppressed by the addition of seeds from the thermodynamic species, enabling us to obtain supramolecular polymers of tuneable length in water for multiple cycles.

## Graphical abstract

Length and pathway control of BODIPY‐based supramolecular polymers in aqueous media has been achieved by programmable hydrogen bonding (intra‐ vs. intermolecular) using a new *N*‐(2‐hydroxyethyl)amide supramolecular synthon as a hydrogen bonding lock. These findings open up new directions in the field of controlled supramolecular polymerization.

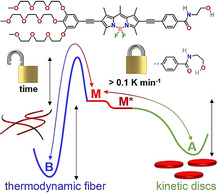

## Introduction

Supramolecular assemblies derived from π‐conjugated scaffolds have attracted widespread attention for optoelectronic applications.^[1]^ In organic electronic materials, the creation of ordered arrays of functionally active molecules is essential for key processes such as charge transport, which in turn determine the final device performance.^[2]^ Therefore, programming the organization of π‐systems across different length scales through high precision self‐assembly is a key but challenging requirement for optimized functional properties.

In recent years, control over the aggregation pathways of various π‐conjugated molecular platforms has allowed the creation of nanostructures with defined dimensions and unique optical and electronic properties.^[3]^ However, a rational understanding of how to control the energy landscape of self‐assembled systems is still challenging, which requires careful selection of experimental variables, optimization of sample preparation protocols, as well as rational monomer design.^[4]^

To date, design strategies for pathway control in self‐assembly have primarily relied on: 1) the length modification of a given molecular fragment;[^4c^, ^4d^, ^4f^, ^4g^, ^4k^] 2) the rational placement of functional groups to trigger additional attractive or repulsive interactions;^[4a,h,j,l,m,w,*x*]^ and 3) the competition between intra‐ and intermolecular hydrogen bonding (H‐bonding).[^4n^, ^4o^, ^4p^, ^4q^, ^4r^, ^4s^, ^4t^, ^4u^, ^4v^, ^4ac^, ^4ad^] The majority of these approaches have been applied to supramolecular systems in organic non‐polar media, where H‐bonding patterns are particularly stable.^[3]^

However, the exploitation of H‐bonding synthons to tune self‐assembly in aqueous medium^[5]^ is considerably more challenging,^[6]^ as competitive interactions with solvent (water) molecules often lead to unexpected outcomes, and therefore, low predictability of the self‐assembly. An additional difficulty is the typically incomplete solubility of amphiphilic molecules in water, making necessary the use of a co‐solvent to enable colloidal stability. In fact, co‐solvents may drastically alter the stability and morphology of aggregates in both non‐polar^[7]^ and aqueous media^[8]^ or even lead to complex energy landscapes by opening up new aggregation pathways. Recent work by Ghosh and co‐workers on aqueous assemblies of napththalene diimide amphiphiles has brought to light that extended H‐bonds can indeed be possible in water when arranged in local hydrophobic environments, leading to assemblies with thermodynamic stability.^[9]^ On the other hand, if water molecules manage to access the functional groups involved in H‐bonding, hydration effects may lead to kinetic trapping of monomers, as recently exploited by Ogi, Yamaguchi and co‐workers to achieve seeded supramolecular polymerization.^[10]^ Even though these strategies have contributed to understand the stability of H‐bonds in water, programming H‐bonding to control the energy landscape of aqueous assemblies still remains elusive.^[11]^

To tackle this issue, we envisaged that rationally placed, conformationally flexible supramolecular synthons^[12]^ may allow the stabilization of different H‐bonding modes, leading to both kinetic and thermodynamic control of self‐assembly processes in water.

**Scheme 1:**
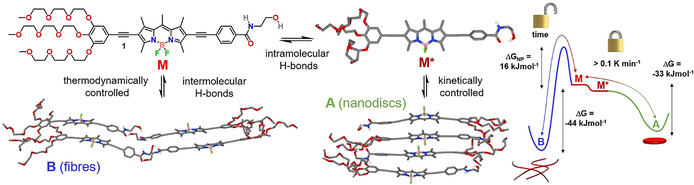
Molecular structure of **1** and representation of its competing aggregation pathways and corresponding energy landscape (thermodynamic parameters obtained by VT‐UV/Vis experiments in water/THF (88/12) at *c*=2.5 μm). M*, **A** and **B** are PM6‐geometry optimized structures.

Inspired by the use of flexible amides to tune competing intra‐ vs. intermolecular H‐bonds in nonpolar media,[^4n^, ^4o^, ^4p^, ^4q^, ^4r^, ^4s^, ^4t^, ^4u^, ^4v^, ^4ac^, ^4ad^] we selected a terminal *N*‐(2‐hydroxyethyl)amide group as new supramolecular synthon and placed it on the 2‐position of a π‐extended amphiphilic BODIPY dye that carries hydrophilic triethylene glycol (TEG) chains on the opposite molecule side (compound **1** in Scheme 1). The choice of a planar and sterically unhindered BODIPY dye as structural motif was made based on the tendency of this class of dyes to readily self‐assemble via π–π interactions.^[13]^ This molecular design is expected to disfavor the intercalation of water molecules into the assembly,[^4l^, ^4m^] allowing for efficient H‐bonding of the *N*‐(2‐hydroxyethyl)amide moieties in a local hydrophobic environment. Furthermore, the possibility of this supramolecular synthon to act as an intramolecularly H‐bonded lock by formation of a seven membered ring may enable kinetic control of the supramolecular polymerization.

Herein, we have used programmable H‐bonding for high‐precision control over the energy landscape and length of supramolecular polymers in aqueous media. Detailed experimental studies complemented by theory reveal that amphiphilic BODIPY **1** initially forms kinetically controlled discoidal H‐type aggregates in water stabilized by an intramolecularly H‐bonded lock involving the *N*‐(2‐hydroxyethyl)amide group. Over time, this kinetic species, which is dominated by strong hydrophobic interactions, slowly transforms into thermodynamically controlled J‐type supramolecular polymers, which involve extended intermolecular H‐bonding in the hydrophobic interior of the fibers. The retarded, cooperative supramolecular polymerization of **1** was ultimately exploited to modulate the fiber length in multiple, subsequent cycles using the seeded‐growth approach. To the best of our knowledge, our results represent the first example of programmable H‐bonding for pathway and length control in aqueous self‐assembly.

## Results and Discussion

**Figure 1:**
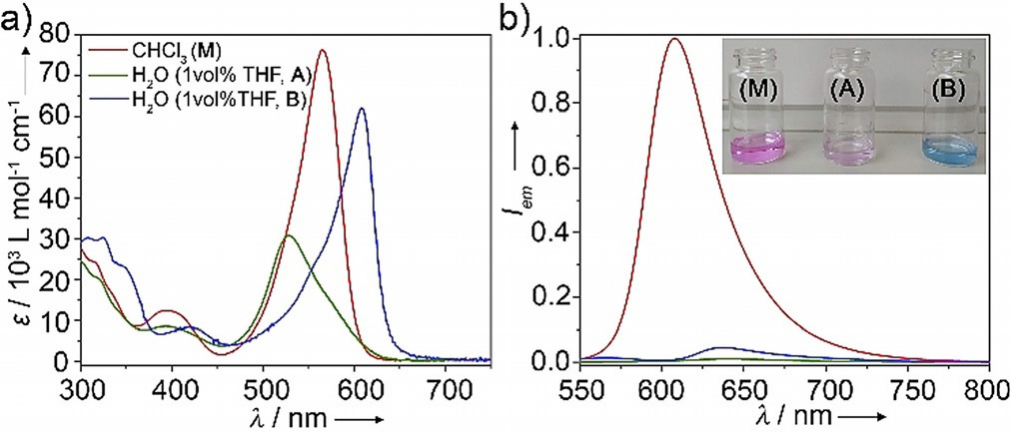
UV/Vis absorption (a) and emission spectra (b) of compound **1** in monomeric (CHCl_3_) and different aggregated states (99 vol % H_2_O / 1 vol % THF, *c*=20 μm) at 298 K (*λ*_exc_=530 for **A** and **M** and 580 nm for **B**).

The synthesis and full characterization of **1** are described in the experimental section of the Supporting Information (S.I.). The UV/Vis spectra of **1** (*c*=20 μm) in a range of organic solvents at room temperature (RT) exhibit the typical spectral pattern of monomeric, non‐aggregated BODIPY dyes, that is, a sharp transition at around 558 nm (S_0_→S_1_) and an additional, less intense band at 397 nm (S_0_→S_2_ transition; Figure 1 a and S1). On the other hand, in aqueous solutions, the main absorption band experiences a strong hypsochromic shift to 530 nm (green spectrum, **A**; Δ*λ*=30 nm). Simultaneously, a weak red shifted shoulder at 576 nm becomes noticeable (Figure 1 a). These spectroscopic features are characteristic of a face‐to‐face stacking (H‐type packing) of the BODIPYs with a slightly twisted arrangement within the assembly.^[14]^ The corresponding solvent‐dependent emission spectra exhibit characteristic sharp monomer bands (*λ*_max_≈605 nm) with moderate‐to‐high quantum yields in “good” solvents such as CHCl_3_ (*φ_F_*=40 %, Figure S1). On the other hand, this emission is remarkably quenched (*φ_F_*=0.9 %) in aqueous media, which is in line with an H‐type aggregation (Figure 1 b).^[15]^

Intriguingly, the previously described blue‐shifted absorption band in water at 530 nm develops over time into a more intense, bathochromically shifted transition centered at 609 nm (Δ*λ*=51 nm, blue spectrum, **B**), which is evident from the color change of the solution from violet to blue (inset of Figure 1 b). In addition to the absorption maximum, both the BODIPY S_0_→S_2_ transition at ca. 400 nm and the bands at 300–330 nm corresponding to the 4‐ethynylphenyl motifs also undergo a bathochromic shift (Figure 1 a). Furthermore, a slight increase of the emission intensity over time in comparison to **A** is noticed (*φ_F_*=1.1 %, Figure 1 b). The overall optical studies suggest that the initially formed H‐type aggregates (**A**) are kinetically controlled and convert over time to a thermodynamically stable J‐type assembly (**B**). The relatively broad absorption band and low emission intensity for **B** might be explained by deviations from an ideal J‐type aggregation, possibly caused by rotational and tilting effects of the BODIPYs upon stacking.

**Figure 2:**
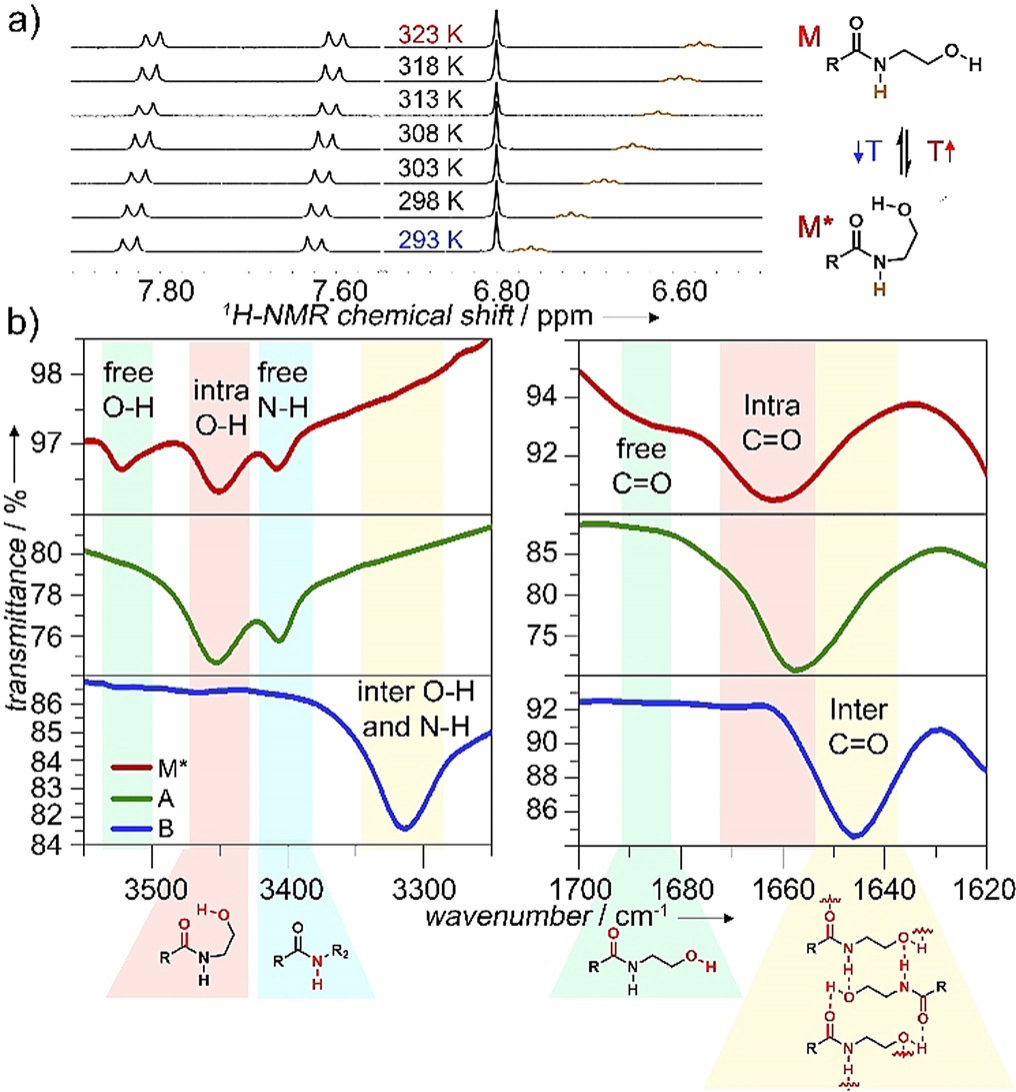
a) VT ^1^H NMR of **1** in CHCl_3_ (0.8 mm, 293–323 K). b) FTIR studies showing characteristic O−H/N−H and carbonyl stretching of M* (0.8 mm in CHCl_3_), **A**, and **B** (thin film from aqueous solution) at 298 K.

Based on these results, we sought to investigate the origin of this aggregate competition, particularly the potential role of the *N*‐(2‐hydroxyethyl)amide group to participate in competing intra‐ vs. intermolecular H‐bonding. To investigate these processes, we initially performed variable temperature (VT) ^1^H NMR studies at 0.8 mm in solvents where **1** preferentially exists in a molecularly dissolved state (CHCl_3_ or DMSO, Figure 2 a and S2). These studies reveal the deshielding of the N−H amide proton upon decreasing the temperature, but no significant shifts for the signals corresponding to the aromatic rings, BODIPY core, or glycol moieties (Figure 2 a and S2). In addition, UV/Vis studies under these conditions (0.8 mm) are nearly similar to those obtained at low concentrations (20 μm) in CHCl_3_ (Figure S2a). A plausible explanation would be the formation of an intramolecularly “locked” H‐bonded monomer species (M*) via a pseudocycle involving the OH group as H‐bonding donor and the amide carbonyl as H‐bonding acceptor, as shown in Scheme 1. The loss in electron density of the carbonyl amide when bound to the hydroxy group might explain the polarization of the amide bond and the resulting deshielding of the adjacent N−H amide protons upon cooling. Unfortunately, the proton of the hydroxy group was not visible even at high temperatures, most likely due to the fast exchange and strong interactions of this group.^[16]^

To further examine the presence of H‐bonds, we investigated solutions of the proposed H‐bonded locked monomer (M*) in chloroform as well as in thin films obtained from aqueous solutions of aggregates **A** and **B** by Fourier‐transform infrared spectroscopy (FTIR, Figure 2 b). For the chloroform solution (where we hypothesize the existence of M*), the carbonyl stretching band appears at 1660 cm^−1^, which corresponds to typical values of moderate‐to‐weak H‐bonding of the carbonyl group.^[17]^ Additionally, a weaker band corresponding to free, non H‐bonded carbonyl groups can also be observed at 1690 cm^−1^ (Figure 2 b, top panel).^[18]^ This relatively small difference in wavenumber between weakly bonded and free carbonyl groups (1660 vs. 1690 cm^−1^) is suggestive of intramolecular H‐bonding.^[17]^ In the O−H/N−H stretching region, three bands at 3521, 3450, and 3407 cm^−1^ are observed for **1** in chloroform (Figure 2 b): whereas the first two bands (at 3521 and 3450 cm^−1^) can be assigned to free and intramolecularly H‐bonded O‐H groups, respectively,^[19]^ the band at 3407 cm^−1^ is a typical value for free, non H‐bonded amide N−H groups.^[20]^ These findings suggest an equilibrium between the open (non‐H‐bonded) and closed, pseudocyclic conformation M* (intramolecular O−H⋅⋅⋅O=C H‐bonds) for **1** in chloroform. The stability of this intramolecular H‐bonded species has been furthermore supported by theoretical calculations showing a heat release (ca. 15 kJ mol^−1^) from open to closed monomer (Scheme 1, Table S4, Figure S3).

We next examined how these H‐bonds would be influenced upon self‐assembly into species **A** and **B**. For kinetic aggregate **A**, the main carbonyl stretching band is positioned nearly at the same wavenumber as that of M* (1660 vs. 1657 cm^−1^), suggesting also the presence of weak, intramolecular H‐bonds for this aggregate (Figure 2 b, middle panel). However, in slight contrast to M*, the band corresponding to free carbonyl groups at 1690 cm^−1^ (which appeared as a shoulder for M*) is absent for **A** as a result of more efficient intramolecular H‐bonding than for M*. This is also supported by the appearance of a single O−H stretching band at 3450 cm^−1^ (corresponding to intramolecularly H‐bonded O−H groups) compared to M*. Also, the fact that the N−H stretching appears at 3407 cm^−1^ indicates that the N−H groups are not involved in H‐bonding. In the light of these observations, we hypothesize that that the carbonyl group in kinetic aggregate **A** is engaged in intramolecular H‐bonds of the type O−H⋅⋅⋅O=C. Due to the similar H‐bonding patterns of **A** and M*, it can be assumed that **A** elongates with consumption of the H‐bonded “locked” monomer (M*).

This behavior differs considerably for the thermodynamic aggregate **B** (Figure 2 b, bottom panel). For this species, the carbonyl stretching is shifted to lower wavenumbers (1646 cm^−1^) compared to **A**, which is typically observed for intermolecular H‐bonding.^[21]^ Interestingly, the O−H stretching band at 3450 cm^−1^ and the N−H stretching band at 3407 cm^−1^ that were observed for aggregate **A** develop into a single band centered at 3365 cm^−1^ for **B** (Figure 2 b, bottom panel). Considering that the O−H stretching bands that were previously assigned to free (3521 cm^−1^) and intramolecularly bonded O−H groups (3450 cm^−1^) are completely absent, this strongly suggests the presence of a stronger involvement of the O−H groups in H‐bonding for **B** compared to **A**. The absence of the amide N−H stretching band at 3407 cm^−1^ points to an involvement of the N−H in H‐bonding, possibly overlapping with the O−H stretching band. The absence of free N−H groups is further supported by FTIR studies of mixtures of **A** and **B** (Figure S2d). Thus, the single band observed for **B** at 3365 cm^−1^ likely originates from contributions of O−H as well as N−H groups being involved in intermolecular H‐bonds with suitable atoms as H‐bonding acceptors.

**Figure 3:**
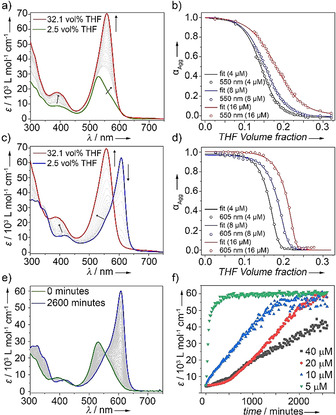
Denaturation UV/Vis Studies of **A** (a) and **B** (b) in water/THF (8 μm, 298 K) and corresponding fits to the denaturation model at various concentrations for **A** (c) and **B** (d). e) Time‐dependent **A**→**B** transformation in water/THF (9:1, v/v) (*c*=10 μm, 1000 rpm) at 25 °C. f) Plot of molar extinction coefficient vs. time at *λ*=610 nm at different concentrations.

To further investigate the mechanistic pathways of **A** (H‐bonding locked) and **B** (intermolecularly H‐bonded), UV/Vis denaturation studies using different ratios of good/poor solvents (THF/water) were carried out at multiple concentrations (4–16 μm, Figure 3 a–d and S4). Addition of a monomer solution of **1** (THF) to an aqueous solution (2.5 vol % THF) of aggregate **A** at the same concentration induces a gradual disassembly process. This can be recognized by the appearance of the characteristic spectral signatures of the monomer species at the expense of **A** (Figure 3 a and S4). Monitoring the absorption changes at 550 nm vs. the volume fraction of THF yields a sigmoidal curve, which is characteristic for an isodesmic polymerization process (Figure 3 b). This mechanism is typically observed for amphiphilic π‐systems that lack directional non‐covalent interaction patterns, such as extended H‐bonding networks.^[22]^ Additionally, aggregates in non‐polar solvents stabilized by intramolecularly H‐bonded pseudocycles are also characterized by an isodesmic mechanism.^[4v]^ Mathematical analysis of these curves by the denaturation model^[23]^ yielded average free Gibbs energy values (Δ*G*_298_) of −44.4 kJ mol^−1^ for **A** (for an overview of the calculated thermodynamic parameters see Table S1).

For the aqueous aggregate solution of **B**, the disassembly is also initiated by addition of the monomer solution of **1** in a good solvent (THF). This process is characterized by the depletion of the sharp J‐band and the concomitant emergence of the monomer band (Figure 3 c and S4). In contrast to **A**, the self‐assembly process is accompanied by a steep transition from monomer to aggregate (Figure 3 d). These non‐sigmoidal curves for **B** are characteristic for a cooperative process, pointing towards the interplay of additional interaction patterns (e.g. extended H‐bonding). This is only possible with consumption of open monomer (**M**), as the carbonyl group of M* is already involved in intramolecular H‐bonds, and therefore unavailable for extended aggregation via intermolecular H‐bonds. The thermodynamic parameters associated with the disassembly of **B** were calculated by fitting the respective experimental data to the denaturation model (Table S1, Figure 2 d), yielding a Δ*G*_298_=−49.1 kJ mol^−1^. These results underline the energetic distinction between both aggregates and suggest an energy gap of Δ*G*_298_≈5 kJ mol^−1^ between **A** and **B**, which may be explained by an additional interaction pattern (e.g. intermolecular H‐bonding) in the self‐assembly process of **B**. This hypothesis is also supported by the cooperative mechanism and FTIR studies.

The higher stability of **B** compared to **A** was additionally proven by thermodynamic analysis of VT absorption and emission studies in THF/water mixtures (Figure S5), which yields a nucleation penalty (Δ*G_NP_*)^[24]^ for **B** of 16.9 kJ mol^−1^ (see Table S2,3). This high Δ*G_NP_* value may explain why the kinetically controlled aggregate **A** is preferentially formed in cooling experiments, regardless of the cooling ramp. A plausible explanation for this behavior would be the absence of a nucleation penalty for **A** due to the isodesmic nature of its self‐assembly.

**Figure 4:**
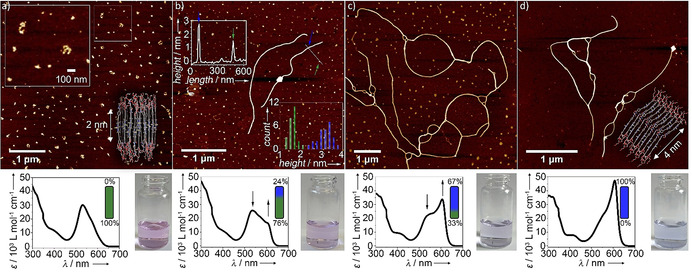
Time‐dependent AFM studies with corresponding UV/Vis spectra and photographs of the solutions used for these studies (8 μm, 1 vol % THF, aged at 298 K): a) 0 days, b) 7 days (inset: section analysis and height profile histogram of nanostructures **A** and **B**), c) 14 days, d) 21 days.

In order to unveil the relationship between both aggregate states, the kinetics of the **A**→**B** transformation in aqueous solutions were examined by time‐dependent absorption studies in a concentration range of 5–40 μm without (Figure S6–S8) and with stirring (Figure 3 e,f and S9–S11). This transformation is relatively slow and occurs in the order of several weeks at room temperature without stirring and using low THF contents (Figure 4). However, the process can be accelerated using higher amounts of THF as a co‐solvent (Figure 3 e,f and S9),^[25]^ mechanical agitation (Figure 3 f and S8b),^[26]^ increasing temperature (Figure S10), and/or even adding seeds of thermodynamic aggregate **B** to kinetically controlled **A** (Figure S6). The decreased lag time for systems with higher amounts of THF imply that hydrophobic interactions are important for the stabilization of **A**. On this basis, the kinetics of this transformation (**A**→**B**) are controlled by the amount of water in the mixture. For example, a complete transformation with 10 vol % THF was achieved after 2 days (20 μm, 1000 rpm, Figure 3 e,f). However, if THF is removed from the solution (see S.I. for details on the experimental method), only traces of **B** (0.02 %) were found in solution after 4 days under the same conditions (Figure S9). This dramatically decreased transformation rate at low or negligible THF contents can be exploited for trapping kinetic species **A**. Monitoring the evolution of the J‐type absorption band of **B** at 610 nm over time at different concentrations revealed increased lag times with increasing concentration, both without (Figure S8) and with stirring (Figure 3 e,f and S11), implying that **A** and **B** are competitive pathways.^[3a]^ This agrees with the former occurring via locked monomer (M*) and the latter via open monomer (**M**).

To further investigate the impact of this extended H‐bonding pattern on the morphology of the assemblies, we have performed time‐dependent atomic force microscopy (AFM, Figure 4, S12, S13). The kinetically controlled state (**A**) is characterized by the formation of clusters of discrete aggregates with diameters of 116±27 nm and a uniform height of 1.6±0.2 nm (Figure 4 a). Given the large difference between the diameter and height values, the nanostructures are likely discoidal in nature. This deviation from an ideal spherical shape is supported by comparison of dynamic (DLS) and static light scattering studies (SLS), where the relation between the hydrodynamic diameter (*D_H_*) and diameter of gyration (*D_g_*) deviates from an ideal spherical shape (*D_g_*=0.77 *D_H_*; Figure S15).^[27]^

Considering the molecular length of the aromatic surface of **1** (2 nm), the discs are likely antiparallel stacks of monomers of **1** that are arranged perpendicular or nearly perpendicular to the mica surface, in order to maximize the interaction of the TEG chains with the hydrophilic substrate.

Combined time‐dependent AFM imaging and UV/Vis experiments using the same solutions were used to monitor the progress of the **A**→**B** transformation. According to UV/Vis, the population of discoidal aggregates decreases to 76 % after 7 days, and few one‐dimensional (1D) fibers can be observed by AFM (Figure 4 b). Height analysis reveals that the height of the 1D fibers is nearly twice (3.1±0.4 nm) that of the discoidal aggregates, pointing to substantial differences in the packing of both aggregates. After 14 days, a further longitudinal growth of the fibers becomes apparent at the expense of the discoidal aggregates, which are reduced in number (33 %, see Figure 4 c). A complete transformation of discs into fibers occurs approximately after 21 days under these conditions, as shown in Figure 4 d. The equilibration of the system is evident from the characteristic J‐type band of **B** observed in UV/Vis experiments, which is in accordance with the exclusive observation of 1D fibers in AFM (Figure 4 d). DLS analysis of this sample reveals, in contrast to aggregate **A**, an angular‐dependent size distribution, underlining the transformation from discrete (**A**) to highly anisotropic (**B**) structures (Figure S15).^[22a]^ Additional SLS studies (system with 1 vol % THF has been investigated) unveil that *D_g_* strongly increases in comparison to *D_H_* during the transformation from **A** (*D_g_*=1.61 *D_H_*) to **B** (*D_g_*=3.53 *D_H_*), which is typically observed in the transformation to long 1D structures.^[27]^ This morphology change can be related to the additional stabilization of **B** via extended H‐bonding.

Considering the multiple types of H‐bonding that the *N*‐(2‐hydroxyethyl)amide group of **1** can establish within **A** and **B**, we investigated various such possibilities by quantum chemical calculations to propose an aggregation model. For kinetically controlled **A**, previous spectroscopic studies had suggested that this assembly originates from intramolecularly locked H‐bonded monomers M*. In addition, discoidal assemblies of unimolecular height were visualized by AFM studies. Based on these considerations, we used as initial guess a tetramer stack of antiparallel‐arranged M* units and optimized the resulting structure by semiempirical calculations at the dispersion‐corrected PM6 level in vacuum.

The optimized tetramer exhibits a face‐to‐face stacking with a large angle between transition dipole moments of adjacent BODIPY dyes (*θ*=67°), as well as extended (intermolecular) aromatic interactions between the intramolecularly locked H‐bonded monomer units M* (Scheme 1 and Figure S16). These observations are in line with the H‐type aggregation experimentally observed. Additional stabilization is gained from the observed antiparallel molecular arrangement, which allows efficient shielding of the water from the hydrophobic core, as often observed in the field of amphiphilic self‐assembly.^[4l]^

**Figure 5:**
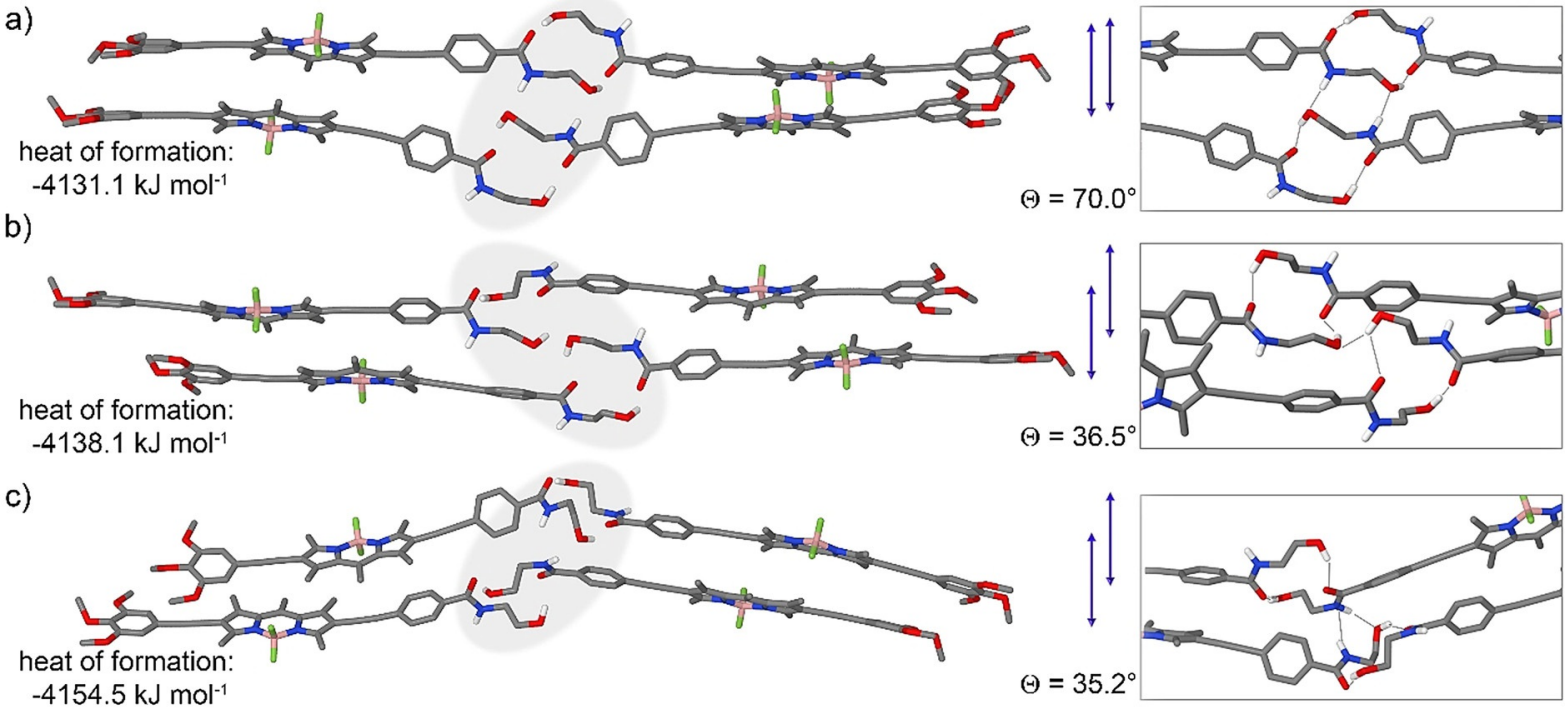
Semiempirical calculations at the dispersion‐corrected PM6 level in vacuum of various possible H‐bonding interactions of assembled structure **B** (TEG chains have been replaced with methoxy chains).

With regards to the thermodynamic species **B**, an approximation was made for the calculations: given the average height of the 1D fibers observed by AFM (3–4 nm), we assumed that the thinnest 1D fibers are formed by stacks of dimers placed side by side. On this basis, we initially optimized a closed dimer that is formed by intermolecular O−H⋅⋅⋅O=C interactions of monomers placed side by side, without any involvement of the N−H group (Figure S17). The dimers of **1** are arranged in a way that the TEG chains are located in the periphery of the 1D fibers while the *N*‐(2‐hydroxyethyl)amide moieties remain in the inner, central part of the stack, as shown in Scheme 1 and in the inset of Figure 4 d. This packing minimizes the exposure of the hydrophobic part of the stack to the aqueous media and ensures that the *N*‐(2‐hydroxyethyl)amide groups are in a more hydrophobic environment where H‐bonding is feasible. Subsequently, to unravel the possible role of the O−H and N−H in extended H‐bonding, two closed dimers were brought to stacking and three resulting tetramers were optimized by semiempirical calculations at the dispersion‐corrected PM6 level in vacuum. Note that the geometry‐optimized tetramers represent only few of the multiple packing possibilities of the monomers of **1** within **B** because of the large number of possible local minima. Interestingly, various types of extended intermolecular H‐bonds were found between the dimers along the stack (Table S4, Figure 5 and S17), which can be most likely explained by the flexibility of the terminal *N*‐(2‐hydroxyethyl)amide group.

The first tetramer optimization unveiled that these closed dimers are prone to further interact by additional weaker intermolecular N−H⋅⋅⋅O−H H‐bonds (Figure 5 a and S17c). However, in this packing, the BODIPYs are stacked with a slip angle (*θ*) of 70°, a typical value observed for H‐type aggregation, and hence, not in agreement with the spectral properties of **B**. A second, alternative geometry‐optimized tetramer exhibits a more pronounced translational offset for the BODIPYs (*θ*=36.5°), where stabilization is gained through intermolecular O−H⋅⋅⋅O−H H‐bonds without any involvement of the amide groups (Figure 5 b and S17d,e). This type of dye arrangement is indeed in agreement with the J‐type exciton coupling experimentally observed for **B**. However, as the IR stretching band corresponding to free N−H groups was completely absent for **B**, this proposed packing is not in full agreement with the experimental results. According to the calculations, the third and most stable geometry‐optimized tetramer exhibits additional weak N−H⋅⋅⋅O−H and N−H⋅⋅⋅N−H interactions, enabling the offset stacking of the BODIPY dyes in a J‐type manner (*θ*=35.2°, Figure 5 c and S17f,g). These interactions would be a reasonable explanation for the broad IR band centered at 3365 cm^−1^ measured for **B**, which was assigned to originate from a combination of weak H‐bonding interactions involving the N−H group and stronger O H⋅⋅⋅O=C interactions. Based on this information, this structure was taken as starting point for the subsequent stack optimization including the TEG chains. Interestingly, a slightly less pronounced translational offset for the BODIPYs (*θ*=50.0°) is observed, possibly due to enhanced intermolecular interactions between the TEG chains.

However, as this slip angle still lies below the magic angle of 54.7° predicted by Kasha's exciton theory, the formation of J‐type aggregates would be feasible (Scheme 1 and Figure S18). This stack is further stabilized by the previously mentioned H‐bonds in the hydrophobic interior, while the glycol chains are pointing to the periphery to maximize their interactions with water molecules from the solvent.

**Figure 6:**
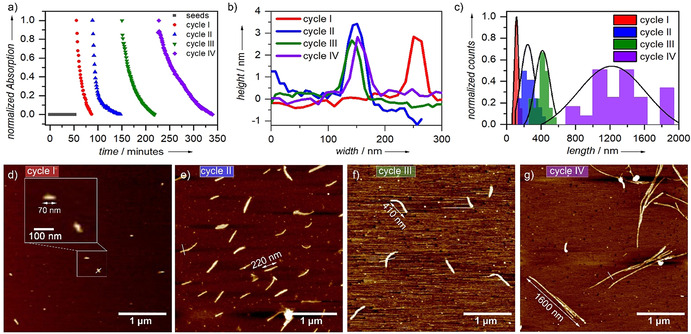
a) Time‐dependent UV/Vis‐studies (data extracted by plotting the wavelength at 530 nm vs. time). b) Height and width analysis of the fibers observed each cycle (data extracted from AFM images) c) Average length distribution of the fibers after each cycle (average obtained by counting 25 fibers). d–g) AFM studies after completion of cycle I (d), II (e), III (f), and IV (g).

As shown previously, mechanistic investigations revealed that the presence of a kinetically controlled H‐bonded locked state, which is dominated by strong hydrophobic effects, retards the spontaneous, cooperative supramolecular polymerization of **1** (**B**) in aqueous medium. These properties prompted us to investigate whether seed‐mediated living supramolecular polymerization (LSP)^[28]^ would be operative in this system. To this end, we initially sonicated the solution of the thermodynamically stable state **B** (1D fibers) for 10 minutes to yield J‐type seeds, which are obtained as small discoidal aggregates (Figures S20, S21). Subsequently, we injected the solution of the kinetically trapped assembly **A** (1 vol % THF in water, *c*=8 μm, 298 K) into the solution of the seeds (**B**) at a ratio of 1:1 and monitored the LSP for multiple cycles by various analytical methods such as UV/Vis, AFM, and DLS (Figure 6; see details in Figures S19–S22). At the beginning of each injection cycle, the characteristic absorption bands of species **A** (at ca. 530 nm) and **B** (at ca. 609 nm) are observed in UV/Vis (Figure S15). Over time, the J‐type band of **B** increases at the expense of the H‐type band of **A**. The consumption of the kinetic species **A** was monitored over time at *λ*=530 nm, revealing a deceleration of the transformation rate upon increasing the number of cycles (Figure 6 a). These observations are in accordance with the dilution of the J‐type seeds, which remain active for further growth over several cycles. Such criteria are essential properties for a system to exhibit LSP. DLS studies disclosed a gradual increase of the hydrodynamic diameter after each cycle, with size distribution maxima ranging from 60 up to 400 nm (Figure S22e). Note that due to the anisotropic nature of **B**, these studies are just a rough approximation to further probe the length changes of the supramolecular polymers in solution.

To ultimately demonstrate length control of the 1D fibers of **B**, we used AFM as an additional tool (Figure 6 c–g, S21, S22, Table S5). The length of 25 fibers was measured each cycle to obtain an average length distribution (Figure 6 c and Table S5). This length analysis supports the stepwise controlled growth after each cycle, ranging from small rod‐like aggregates with an average size of 111±24 nm after the first cycle up to fibers with a uniform length of 1.2±0.4 μm after the fourth cycle (Figure 6 c–g, S21, S22, Table S5). While the length increases each cycle, the height (ca. 3 nm) and width (ca. 65 nm) of the fibers remain almost unaltered (Figure 6 b). In contrast to the uncontrolled growth in the absence of seeds (see Figure 4), shorter, but more distinct structures are found (Figure 6). This phenomenon can be explained by the addition of a fixed amount of seeds (1:1), which help to consume the kinetic species in a controlled fashion. However, without the addition of seeds, only minor amounts of active ends of **B** are present in solution in comparison to kinetic state **A**. As a result, larger fibers with a tendency to form circular loops are observed. Our system, apart from being one of the few examples where length control of supramolecular polymers is achieved in aqueous media, also covers small fiber ranges, that is, 111 nm after the first cycle.

## Conclusion

In conclusion, we have exploited a switchable H‐bonding lock as new strategy for pathway and length control of supramolecular polymers in aqueous media. To analyze this effect, we designed a new amphiphilic, π‐extended BODIPY derivative **1** with hydrophilic TEG chains and an additional flexible *N*‐(2‐hydroxyethyl)amide synthon placed on the opposite molecule side. Compound **1** initially forms kinetically controlled discoidal H‐type aggregates (**A**) in aqueous media via an intramolecularly locked, H‐bonded pseudocycle formation involving the terminal OH group and the amide carbonyl of the *N*‐(2‐hydroxyethyl)amide group. Keeping an aqueous solution (with minor amounts of THF) of **A** at room temperature for several weeks leads to a transformation into thermodynamically controlled fibrillar J‐type aggregates **B**, which, in contrast to **A**, are stabilized by extended intermolecular H‐bonds. Kinetic studies at different concentrations reveal that **A** and **B** are competitive pathways, that is, interconversion is only possible via monomer formation. Furthermore, studies with different percentages of co‐solvent (THF) in water reveal that the kinetics of the **A**→**B** transformation are governed by the hydrophobic effect, as addition of THF destabilizes **A** and accelerates the transformation to **B**. VT and denaturation studies by absorption and emission spectroscopy disclose an isodesmic aggregation process for **A** and a cooperative supramolecular polymerization for **B**. The stability of kinetic aggregate **A** in water (several weeks at room temperature) can be explained by the high nucleation penalty of cooperative path **B**. Interestingly, the seeded‐growth approach could be applied to achieve supramolecular polymers in water with tuneable length for multiple cycles, thereby broadening the scope of living supramolecular polymerization. Thus, programmable H‐bonds (intra‐ vs. intermolecular) have been exploited for the first time for pathway and length control of supramolecular polymers in aqueous media. We believe this approach will enrich and open new directions in the area of controlled supramolecular polymerization in aqueous media.

## Conflict of interest

There are no conflicts to declare.

## Supporting information

As a service to our authors and readers, this journal provides supporting information supplied by the authors. Such materials are peer reviewed and may be re‐organized for online delivery, but are not copy‐edited or typeset. Technical support issues arising from supporting information (other than missing files) should be addressed to the authors.

Supplementary
