# Supplementary material for: Pathway and Length Control of Supramolecular Polymers in Aqueous Media via a Hydrogen Bonding Lock
Source: Angew Chem Int Ed Engl. 2020 Dec 22;60(8):4368–76. doi: 10.1002/anie.202012710 (PMC7898687; doi:10.1002/anie.202012710)
Supplement: Supplementary file 1 — Supplementary [file ANIE-60-4368-s001.pdf]

Angewandte Chemie
Eine Zeitschrift der Gesellschaft Deutscher Chemiker

Supporting Information

Pathway and Length Control of Supramolecular Polymers in Aqueous Media via a Hydrogen Bonding Lock

*Ingo Helmers, Goutam Ghosh, Rodrigo Q. Albuquerque, and Gustavo Fernández**

anie_202012710_sm_miscellaneous_information.pdf

Table of Contents

Table of Contents 2
Experimental Procedures 3
Materials and Methods 3
Synthetic details and characterization 4
Results and Discussion 9
Isodesmic Model 9
Temperature-dependent Isodesmic Model 9
Nucleation-Elongation model for Cooperative Supramolecular Polymerizations 10
Denaturation model 11
Thermodynamic Parameters 12
Fluorescence Quantum Yields 12
Semiempirical calculations at the dispersion-corrected PM6 level in vacuum 12
Supplementary Figures 13

Experimental Procedures

Materials and Methods

Chemical and Reagents: All chemicals were purchased from Sigma-Aldrich (St. Louis, MO, USA), TCI Europe N.V. (Tokyo, JP) or Alfa Aesar (Ward Hill, MA, USA), with minimum analytical grade quality, and used without further purification unless otherwise stated. Dichloromethane was first pre-dried over $CaCl_2$ and then distilled over P_2O_5 in an argon atmosphere. Silica gel was used for column chromatography unless otherwise indicated.

Column chromatography. Preparative column chromatography was conducted in self-packed glass columns of different sizes with silica gel (particle size: 40 – 60 µm, *Merck*). Dichloromethane was distilled before usage.

NMR spectroscopy: ^1H and ^{13}C NMR spectra were recorded at 298 K on Avance II 300 and Avance II 400 from Bruker for routine experiments using tetramethylsilane (TMS) as internal reference, and DD2 500 and DD2 600 from Agilent for characterization purposes. Multiplicities for proton signals are abbreviated as s, d, t, q and m for singlet, doublet, triplet, quadruplet and multiplet respectively.

Mass spectrometry: ESI mass spectra were measured with a Bruker MicrOToF system.

UV/Vis and fluorescence spectroscopy: UV/Vis absorption spectra were recorded on a Jasco V-770 or a Jasco V-750 spectrophotometers, both equipped with peltier cells and Julabo F250 water circulation units. Fluorescence spectra were recorded on a Jasco FP-8500 spectrofluorometer equipped with the same water circulation unit.

FT-IR spectroscopy: Solution state and solid-state FT-IR was measured using a JASCO-FT-IR-6800 using a CaF_2 cell with a path length of 0.1 mm. For all measurements, spectroscopic grade solvents (UVasol) from Merck were used.

AFM: The AFM images have been recorded on a Multimode®8 SPM Systems manufactured by Bruker AXS. The used cantilevers were AC200TS by Oxford Instruments with an average spring constant of 9 N m^{-1} , an average frequency of 150 kHz, an average length of 200 µM, an average width of 40 µM and an average tip radius of 7 nm. All solutions have been spin-coated onto a mica surface using a spin rate between 2000 and 4000 rpm.

Dynamic and Static Light Scattering: DLS and SLS measurements were performed on a CGS-3 Compact Goniometer System from ALV, equipped with a LSE-5004 Light Scattering Electronics (22 mW HeNe Laser (633 nm)) and Multiple Tau Digital Correlator unit from ALV. Solvents were filtered prior to sample preparation through nylon or Teflon filters with a pore size of 0.45 µm. The Experimental SLS data was analyzed with help of the ALV-Fit & Plot application.

Sample Preparation Method: In general, the compound was dissolved in THF and subsequently water was added to obtain aggregate **A**. Furthermore, we prepared samples where the aliquots of THF (1 vol%) were removed. This was achieved by evaporating the solvent mixture at 50 mbar, 10 rpm for 40 minutes at 298 K on a rotary evaporator. Low temperature and low spinning rates have to be applied to prevent the formation of aggregate species **B**. The solutions have been weighted before and after evaporation and refilled with the missing amount of water to keep the concentration constant. The weight loss was ~3% after evaporating solution with 1 vol% of THF, therefore most likely also a minor amount of water was removed from these solutions.
Aggregate **A** transforms over time to aggregate **B**. The transformation time can be accelerated by applying mechanical agitation, increasing the temperature and/or the co-solvent ratio (increasing percentages of good solvent: THF).

SUPPORTING INFORMATION

Synthetic details and characterization

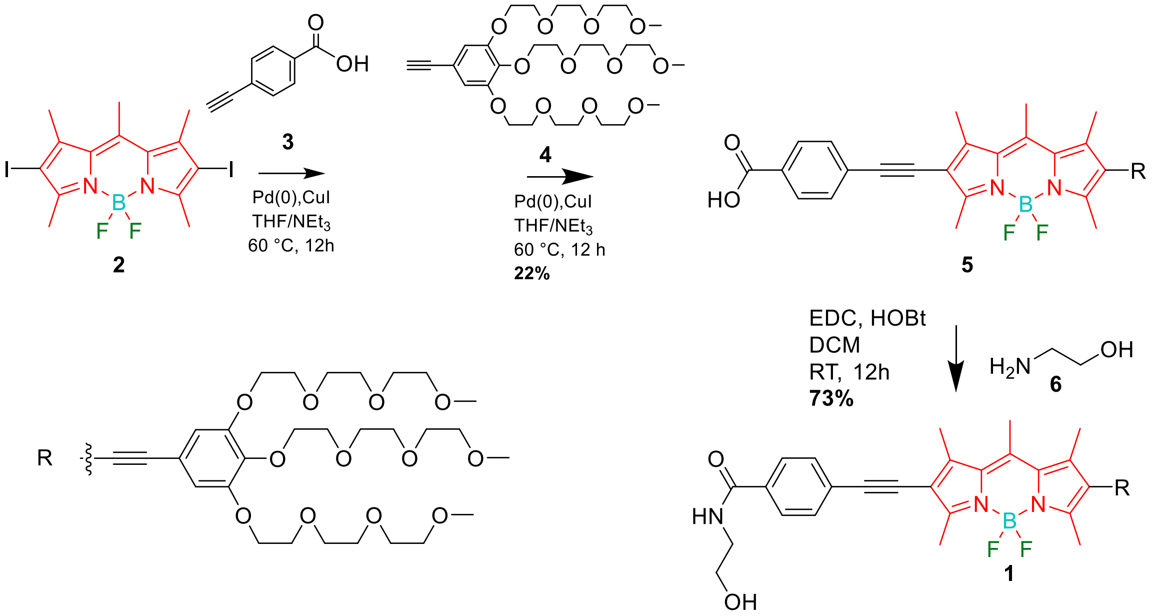

5-Difluoro-2,8-diiodo-1,3,7,9,10-pentamethyl-5H-4l4,5l4-dipyrrolo[1,2-c:2',1'-f][1,3,2]diazaborinine (**2**), [1] 4-ethynylbenzoic acid (**3**), [2] 5-ethynyl-1,2,3-tris(2-(2-(2-methoxyethoxy)ethoxy)ethoxy)benzene (**4**)[3] were prepared following reported synthetic procedures and showed identical spectroscopic properties to those reported therein.

---

[1] A. Rödle, B. Ritschel, C. Mück-Lichtenfeld, V. Stepanenko, G. Fernández, *Chem. Eur. J.* **2016**, DOI: 10.1002/chem.201602592.
[2] F. A. Mandl,V. C. Kirsch,I. Ugur,E. Kunold, J. Vomacka,C. Fetzer,S. Schneider,K. Richter,T. M. Fuchs,I. Antes and S. A. Sieber, *Angew. Chem. Int. Ed.* **2016**, *55*, 14852 –14857.
[3] S. Chen, X. Huang, S. Decurtins, C. Albrecht, S. Liu, *Polyhedron* **2017**, *134*, 287–294.

Synthesis of 4-((5,5-difluoro-1,3,7,9,10-pentamethyl-8-((3,4,5-tris(2-(2-(2-methoxyethoxy)-ethoxy)ethoxy)phenyl)ethynyl)-5*H*-5λ[4],6λ[4]-dipyrrolo[*1,2-c:2',1'-f*][*1,3,2*]diazaborinin-2-yl)ethynyl)benzoic acid (5)

5,5-difluoro-2,8-diiodo-1,3,7,9,10-pentamethyl-5*H*-4λ4,5λ4-dipyrrolo[1,2-c:2',1'-f][1,3,2] diazaborinine (1, 2.11 g, 4.11 mmol, 1 eq), $(PPh_3)_4Pd$ (0.24 g, 0.21 mmol, 0.05 eq) and CuI (39.0 mg, 0.21 mmol, 0.05 eq) were dissolved after three vacuum-argon cycles in anhydrous THF (15 mL) and NEt_3 (15 mL). The reaction mixture was heated to 50 °C and 4-ethynylbenzoic acid (2, 0.60 g, 4.11 mmol, 1 eq) dissolved in THF (150 mL) was added dropwise over the course of 5 hours to the solution and stirred for additional 12 h at 50°C. The mixture was concentrated, the residue dissolved in DCM (150 mL), precipitated with *n*-Pentane (150 mL) and washed (3 x 150 mL) with DCM/*n*-Pentane (1/1, (*v*/*v*)). The crude product (0.78 g, 1.47 mmol) was used without further purification within the next reaction.
The raw product (0.78 g, 1.47 mmol, 1 eq), $(PPh_3)_4Pd$ (84.6 mg, 73.2 µmol, 0.05 eq) and CuI (14.0 mg, 73.2 µmol, 0.05 eq) were dissolved after three vacuum-argon cycles in anhydrous Dioxane (20 mL) and $HNiPr_2$ (10 mL). The reaction mixture was heated to 50 °C and 5-ethynyl-1,2,3-tris(2-(2-(2-methoxyethoxy)ethoxy)ethoxy)benzene (3, 0.67 g, 1.47 mmol, 1 eq) was added. The solution was stirred for 12 h, acidified with HCl (2M) to pH 1 and extracted with DCM (3 x 150 mL). The organic phase was dried over Na_2SO_4, the solvent removed and the crude product purified by silica gel column chromatography using a gradient from DCM to DCM/MeOH (8/2, (*v*/*v*)) as eluent. Finally, a reverse phase (C18) column chromatography using a gradient from water/ACN (9/1, (*v*/*v*)) to ACN as eluent was performed.
Yield: 0.90 g (0.90 mmol, 22%) of a dark purple solid.

Characterization:

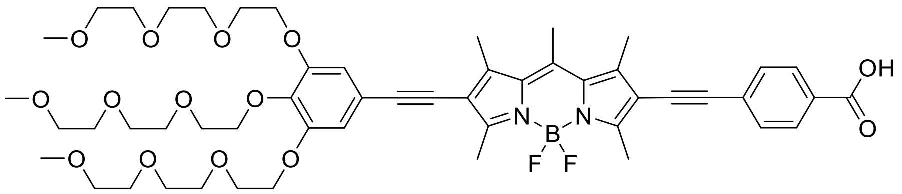

Chemical Formula: $C_{52}H_{67}BF_2N_2O_{14}$
Exact Mass: 992.4653
Molecular Weight: 992.9148

^{1}H NMR (300 MHz, $CDCl_3$):
δ (in ppm) = 7.99 (d, *J* = 8.0 Hz, 2H-Ar-COOH), 7.56 (d, *J* = 8.0 Hz, 2H-Ar-COOH), 6.72 (s, 2H-Ar-TEG), 4.21 (m, 6H-C-O-Ar), 4.00 – 3.66 (m, 24H-TEG), 3.64 – 3.55 (m, 6H-TEG), 3.41 (m, 9H-CH_3), 2.71 (s, 6H-BODIPY), 2.61 (s, 3H-BODIPY), 2.54 (s, 3H-BODIPY), 2.49 (s, 3H-BODIPY).

^{13}C NMR (75 MHz, $CDCl_3$):
δ (in ppm) = δ 168.8, 157.1, 155.8, 152.5, 142.9, 142.5, 142.2, 139.2, 132.2, 131.7, 130.9, 129.9, 129.2, 128.2, 118.3, 116.4, 115.1, 110.9, 96.7, 96.1, 85.0, 80.7, 72.5, 72.0, 72.0, 70.8, 70.8, 70.7, 70.6, 70.6, 70.5, 69.7, 68.9, 59.1, 59.1, 16.8, 16.2, 15.6, 13.7, 13.6.

ESI-MS (TOF):
m/*z* 991.45894 $[M-H]^-$, calculated for $C_{52}H_{66}N_2O_{14}BF_2$: 991.45580

Synthesis of 4-((5,5-difluoro-1,3,7,9,10-pentamethyl-8-((3,4,5-tris(2-(2-(2-methoxyethoxy)ethoxy)ethoxy)phenyl)ethynyl)-5*H*-4λ[4],5λ[4]-dipyrrolo[*1,2-c:2',1'-f*][*1,3,2*]diazaborinin-2-yl)ethynyl)-*N*-(2-hydroxyethyl)benzamide (1)

4-((5,5-difluoro-1,3,7,9,10-pentamethyl-8-((3,4,5-tris(2-(2-(2-methoxyethoxy)ethoxy)ethoxy)phenyl)ethynyl)-5*H*-4λ[4],5λ[4]-dipyrrolo[1,2-c:2',1'-f][1,3,2]diazaborinin-2-yl)ethynyl)benzoic acid (131 mg, 0.13 mmol, 1 eq), HOBt (17.7 mg, 0.13 mmol, 1 eq) and EDC (25.1 mg, 0.13 mmol, 1 eq) were dissolved in anhydrous DCM (10 mL) and stirred for 2 hours. 2-aminoethane-1-ol (24.1 mg, 0.39 mmol, 3 eq) was added to the reaction mixture and the reaction was stirred for further 10 hours. The solvent was removed and the crude product purified by silica gel column chromatography using a gradient from DCM to DCM/MeOH (10 vol%) as eluent. Finally, a reverse phase (C18) column chromatography using a gradient from water/ACN (9/1, (*v*/*v*)) to ACN as eluent was performed
Yield = 98 mg (0.10 mmol, 73%) of a purple solid.

Characterization:

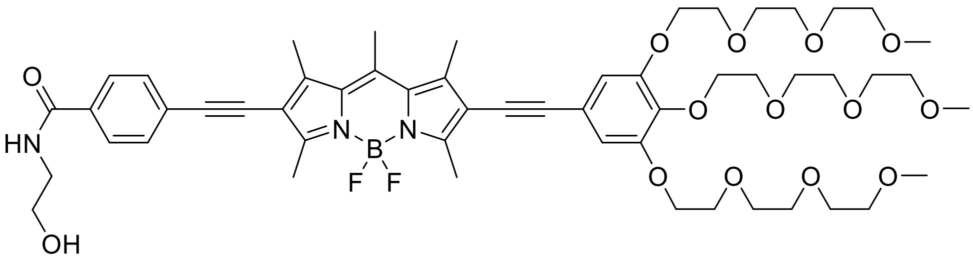

Chemical Formula: $C_{54}H_{72}BF_2N_3O_{14}$
Exact Mass: 1035,51
Molecular Weight: 1035,98

^{1}H NMR (500 MHz, CD_2Cl_2):
δ (in ppm) = 7.73 (d, *J* = 8.2 Hz, 2H), 7.53 (d, *J* = 8.2 Hz, 2H), 6.97 (t, *J* = 5.6 Hz, 1H), 6.73 (s 2H), 4.16 (m, 6H), 4.01 – 3.48 (m, 30H), 3.35 (m, 9H), 2.68 (s, 6H), 2.61 (s, 3H), 2.55 (s, 3H), 2.52 (s, 3H).

^{13}C NMR (126 MHz, CD_2Cl_2):
δ (in ppm) = 167.9, 157.3, 156.6, 153.0, 143.8, 143.1, 142.9, 139.4, 134.1, 132.7, 131.7, 127.7, 127.1, 118.9, 116.7, 115.9, 111.1, 97.1, 96.4, 84.6, 81.2, 73.0, 72.5, 72.4, 71.3, 71.1, 71.1, 71.0, 70.2, 69.3, 62.7, 59.2, 43.7, 30.3, 17.5, 16.5, 16.3, 14.0, 13.9.

ESI-MS (TOF)**:**
m/*z* 1058.49704 $[M+Na]^+$, calculated for $C_{54}H_{72}N_3O_{14}BF_2$ Na: 1058.49767

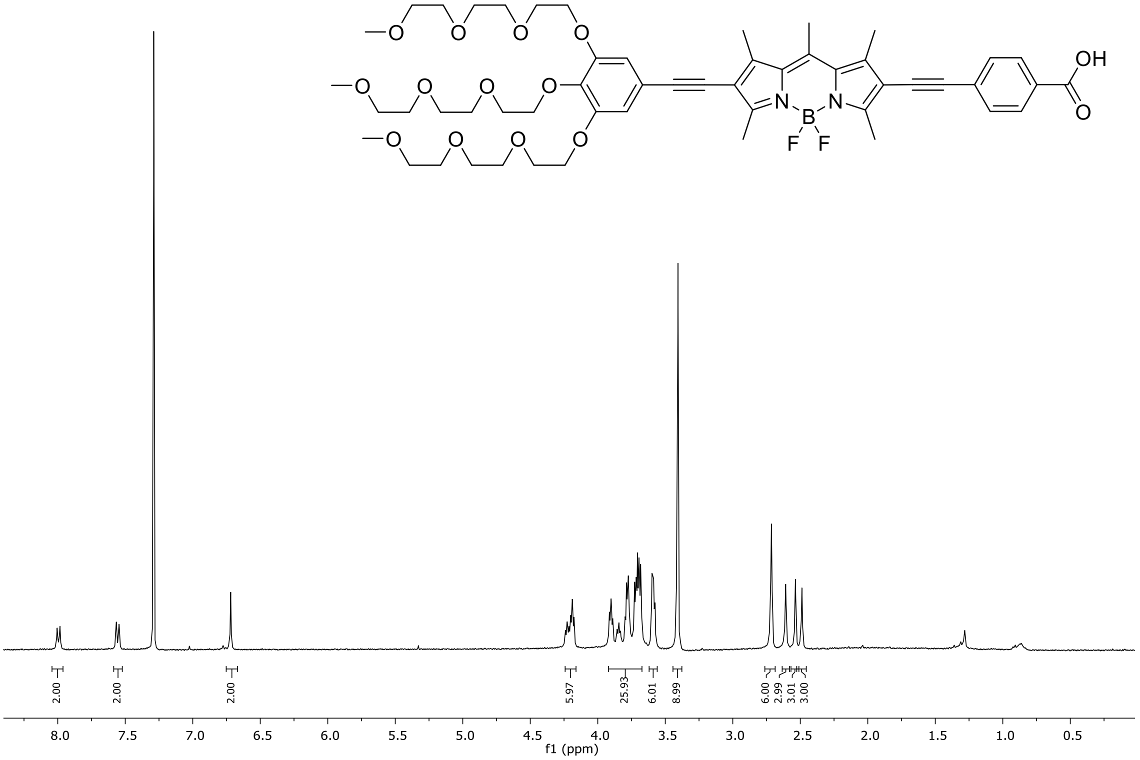

Figure SE1. ^{1}H NMR (400 MHz, $CDCl_3$, 298 K) of **5**.

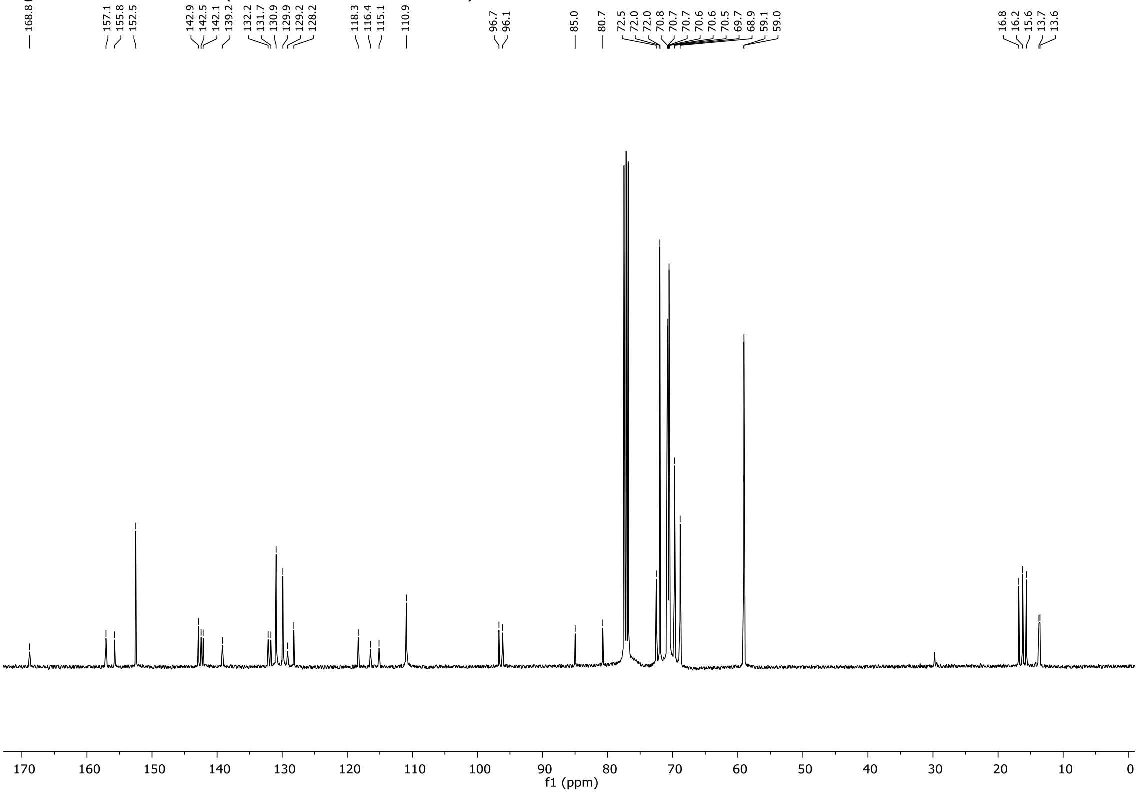

Figure SE2. ^{13}C NMR (101 MHz, $CDCl_3$, 298 K) of compound **5**.

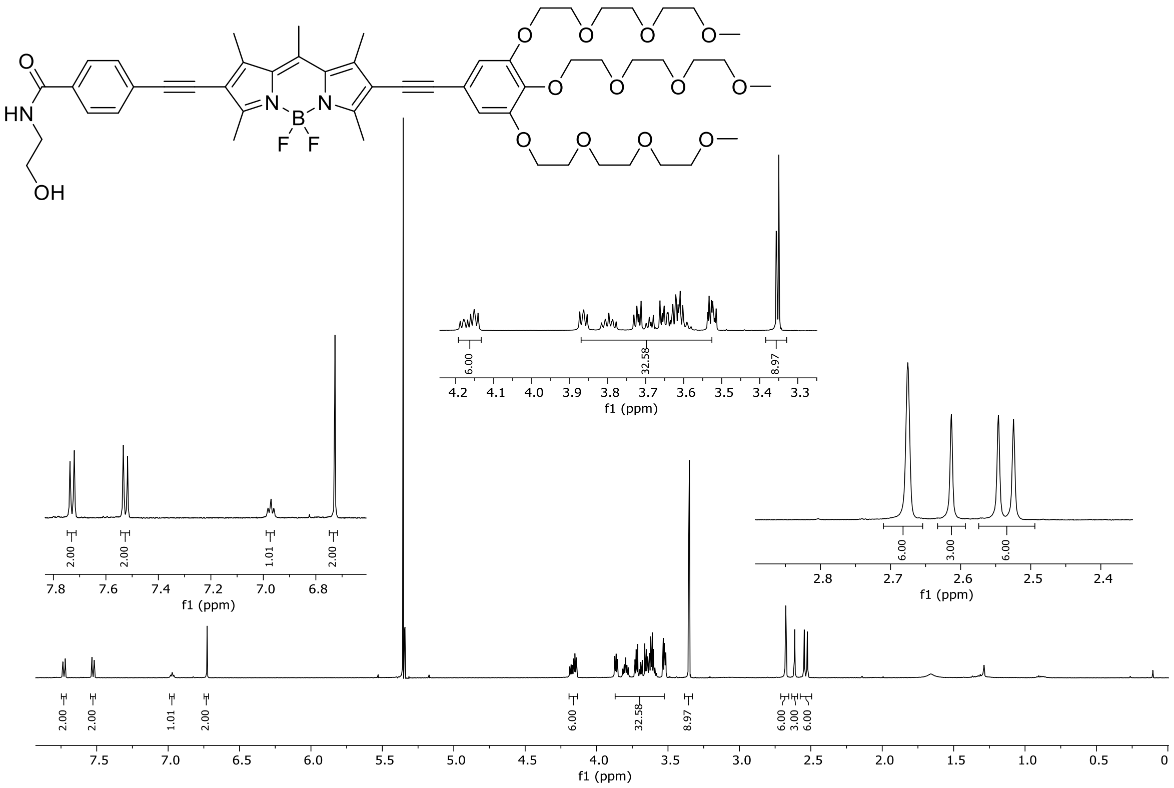

Figure SE3. ^{1}H NMR (500 MHz, CD_2Cl_2, 298 K) of **1**.

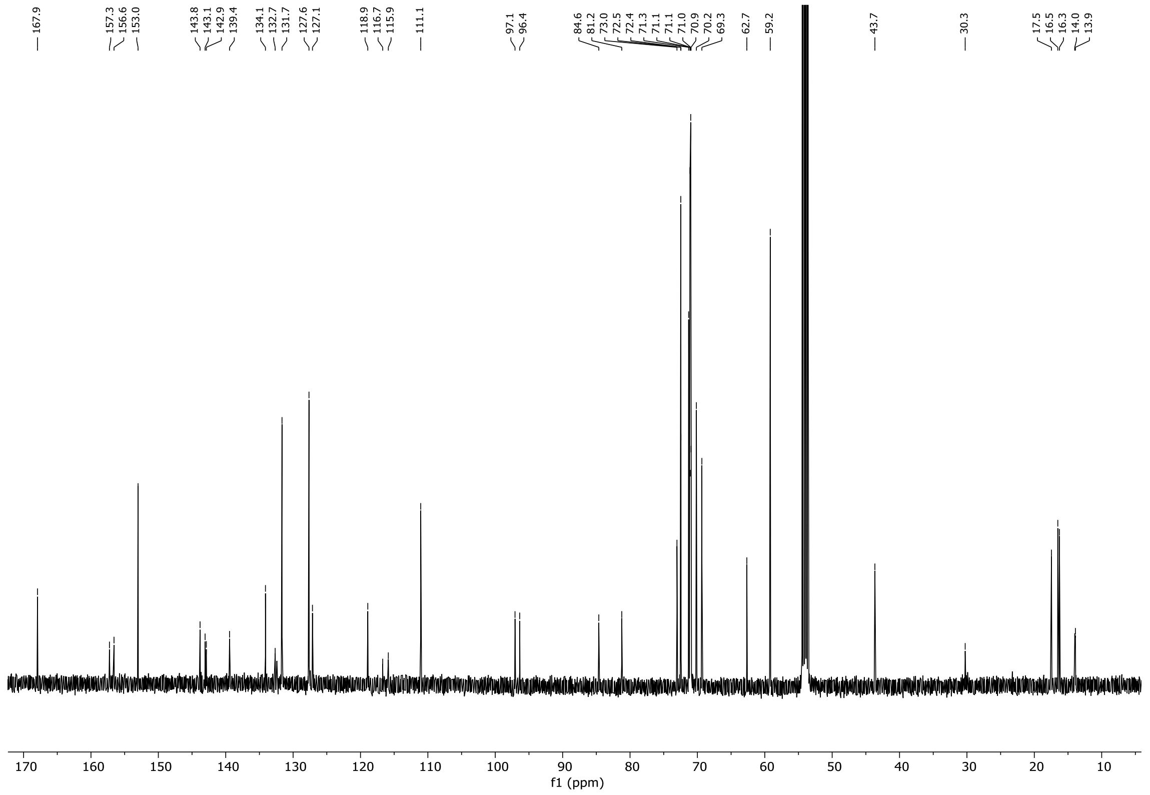

Figure SE4. ^{13}C NMR (126 MHz, CD_2Cl_2, 298 K) of compound **1**.

Results and Discussion

Isodesmic Model[4]

The self-assembly of **A** is described using the isodesmic model. This model assumes that the aggregates are noncyclic and one dimensional. The reversible non-covalent binding doesn't occur any change for each growing step therefore the reactivity of the end groups is equivalent during this process. According to that the equilibrium constants and Gibbs free energy changes are identical for each step, too.

Temperature-dependent Isodesmic Model.

The temperature-dependent data was fitted to the isodesmic model by using the Boltzmann equation.

$$\varepsilon = \varepsilon_{mon} + \frac{\varepsilon_{agg} - \varepsilon_{mon}}{1 + e^{\frac{\mathrm{T-T_m}}{\mathrm{T^*}}}} \qquad (\mathbf{1})$$

ε_{mon} = molar extinction coefficient of monomer species
ε_{agg} = molar extinction coefficient of aggregate species
T_m= melting Temperature
T*= characteristic temperature that is related to the slope of the function at $T = T_m$

$$\alpha_{\mathrm{agg}}(\mathrm{T}) = \frac{\varepsilon - \varepsilon_{mon}}{\varepsilon_{agg} - \varepsilon_{mon}} \qquad (\mathbf{2})$$

By changing equation **1** and inserting the degree of polymerization or molar fraction $\alpha_{agg}(T)$ (**2**), equation **3** can be obtained which shows the sigmoidal relation from the degree of polymerization to the temperature. The values of α_{agg}(T) range between 0 and 1, when the factor reaches 0.5 the temperature can be denoted as the melting Temperature T_m.

$$\alpha_{\mathrm{agg}}(\mathrm{T}) \cong \frac{1}{1 + e^{\frac{\mathrm{T-T_m}}{\mathrm{T^*}}}} \qquad (\mathbf{3})$$

The characteristic temperature T^* is related to the slope of the function **3** at $T = T_m$. The enthalpy release ΔH can be obtained with the characteristic temperature T^* *via:*

$$\left|\frac{\delta\alpha_{agg}}{\delta T}\right|_{T=T_m} = \frac{-1}{4T^*} \approx \frac{\left(\frac{\sqrt{2}-1}{2\sqrt{2}-1}\right)\Delta H}{RT_m^2} \qquad (\mathbf{4})$$

By changing equation **4** T^* can be written as a function of ΔH and T_m:

$$T^* \cong \frac{-RT_m^2}{0.908\Delta H} \qquad (\mathbf{5})$$

To obtain molar fraction of aggregated species $\alpha_{agg}(T)$ as a function of the temperature T the equation **3** and **5** are combined:

$$\alpha_{\mathrm{agg}} \cong \frac{1}{1 + \exp\left(-0.908\Delta\mathrm{H}\frac{\mathrm{T-T_M}}{\mathrm{RT_M^2}}\right)} \qquad (\mathbf{6})$$

[4] M. M. Smulders, M. M. L. Nieuwenhuizen, T. F. A. De Greef, P. Van der Schoot, A. P. H. J. Schenning, E. W. Meijer, *Chem. Eur. J.* **2010**, *16*, 362-367; T. F. A. De Greef, M. M. J. Smulders, M. Wolffs, A. P. H. J. Schenning, R. P. Sijbesma, E. W. Meijer, *Chem. Rev*. **2009**, *109*, 5687-5754.

The number-averaged degree of polymerization $DP_N(T)$ is obtained from $\alpha_{agg}(T)$ *via*:

$$DP_N = \frac{1}{\sqrt{1-\alpha_{agg}(T)}} \qquad (\mathbf{7})$$

The degree of polymerization is related to the equilibrium constant by equation **8**.

$$\alpha_{agg} = 1 - \frac{2Kc_T + 1 - \sqrt{4K(T)c_T + 1}}{2K^2 c_T^2} \qquad (\mathbf{8})$$

Equation **7** and **8** combined, depict the Relationship of DP_N to the equilibrium constant *K* and the total concentration of molecules c_T via:

$$DP_N = \frac{1}{\sqrt{1-\alpha_{agg}(T)}} = \frac{1}{2} + \frac{1}{2}\sqrt{4K(T)c_T + 1} \qquad (\mathbf{9})$$

This equals:

$$DP_N = \frac{c_T}{c_N} = \frac{c_T(1-Kc_1)}{c_1} = \frac{1+\sqrt{4Kc_T+1}}{2} \qquad (\mathbf{10})$$

Nucleation-Elongation model for Cooperative Supramolecular Polymerizations

The equilibrium between the monomeric and supramolecular species can be described in a cooperative process with the *Nucleation-Elongation model* developed by Ten Eikelder, Markvoort and Meijer[5,6]. This model is used to describe the aggregation of **B** which exhibits a non-sigmoidal cooling curve, as shown in the fluorescence and UV-Vis temperature-dependent experiments. The model extends nucleation-elongation based equilibrium models for growth of supramolecular homopolymers to the case of two monomer and aggregate types and can be applied to symmetric supramolecular copolymerizations, as well as to the more general case of nonsymmetric supramolecular copolymerizations.

In a cooperative process, the polymerization occurs by a nucleation step, with a nucleus size assumed of 2, and a following elongation step. The values T_e, $\Delta H°_{nucl}$, $\Delta H°$ and $\Delta S°$ can be found by a non-linear least-square analysis of the experimental melting curves. The equilibrium constants associated to the nucleation and elongation phases can be calculated using equations **11** and **12**:

Nucleation step: $$K_n = e^{\left(\frac{-((\Delta H^o - \Delta H^o_{NP}) - T\Delta S^o)}{RT}\right)} \qquad (\mathbf{11})$$

Elongation step: $$K = e^{\left(\frac{-(\Delta H^o - T\Delta S^o)}{RT}\right)} \qquad (\mathbf{12})$$

And the cooperativity factor (σ) is given by:

$$\sigma = \frac{K_n}{K_e} = e^{\left(\frac{\Delta H^o_{NP}}{RT}\right)} \qquad (\mathbf{13})$$

---

[5] H. M. M. Ten Eikelder, A. J. Markwoort, T. F. A. De Greef, P. A. J. Hilbers, *J. Phys. Chem. B* **2012**, *116*, 5291-5301.
[6] A. J. Maarkvort, H. M. M. Ten Eikelder, P. J. J. Hilbers, T. F. A. De Greef, E. W. Meijer, *Nat. Commun.* **2011**, *2*, 509-517.

Denaturation model [7]

The denaturation model is based on the concentration-dependent supramolecular polymerization equilibrium model by Goldstein,[8] whereas the polymerization is described as a sequence of monomer addition equilibria.

$$[P_n] = K_n[P_{n-1}][X]$$

$$[P_{n+1}] = K_e[P_n][X]$$

$$[P_i] = K_e[P_{i-1}][X]$$

For the cooperative model K_n<K_e and for the isodesmic process K_n=K_e. The concentration for each species P_i is given by $[P_i] = K_n^{i-1}[X]^i$ for $i \leq n$ and $[P_i] = K_e^{i-n}K_n^{n-1}[X]^i$ for $i > n$.

The dimensionless mass balance is obtained by inserting the dimensionless concentration $p_i = K_e[P_i]$, monomer concentration $x = K_e[X]$ and concentration of each species P_i (for $i \leq n$) : $p_i = \sigma^{i-1}x^i$ and for $i > n$: $p_i = \sigma^{n-1}x^i$):

$$x_{tot} = \sigma^{-1}\sum_{i=1}^{n} i(\sigma x)^i + \sigma^{n-i}\sum_{i=n+1}^{\infty} ix^i.$$

Both sums are evaluated by using standard expressions for converging series:

$$x_{tot} = \left(\frac{(\sigma x)^{n+1}(n\sigma x - n - 1)}{(\sigma x - 1)^2} + \frac{\sigma x}{(\sigma x - 1)^2}\right) - \sigma^{n-1}\left(\frac{x^{n+1}(nx - n - 1)}{(x - 1)^2}\right)$$

With $x_{tot} = c_{tot}K_e$ and $c_{tot:}$ the total monomer concentration

The sum solved by standard numerical methods (Matlabfzerosolver) results in the dimensionless monomer concentration x. Considering that every species with $i > 1$ is defined as aggregate, the degree of aggregation results in:

$$\varphi = \frac{x_{tot} - x}{x_{tot}}$$

Via $K_e = \exp(-\frac{\Delta G^0}{RT})$ the denaturation curves can be obtained with f defined as volume fraction of good solvent:

$$\Delta G^{0,} = \Delta G^0 + m\,f$$

It is assumed that the cooperativity factor σ is independent of the volume fraction and the m value involved in the elongation equals the m value involved in the nucleation.
The denaturation data need to be transformed into the normalized degree of aggregation, if fitted to the supramolecular polymerization equilibrium model:

$$(f) = \frac{A(f) - A(f = 0)}{A(f = 1) - A(f = 0)}$$

The optimization of the four needed parameters (ΔG_0*, m, σ and p*) to fit the equilibrium model to the experimental data (normalized degree vs *f*) is done by the non-linear least-squares analysis using Matlab (lsqnonlinsolver). The data is then fitted with the non-linear least squared regression (Levenberg-Marquardt algorithm).

[7] P. A. Korevaar, C. Schaefer, T. F. A. de Greef, E. W. Meijer, *J. Am. Chem. Soc.*, **2012**, *134 (32),* 13482–13491.
[8] Goldstein, R. F.; Stryer, *L. Biophys. J.*, **1986**, *50*, 583-599.

Thermodynamic Parameters

The thermodynamic Parameters for **A** and **B** (**Table S1-3**) were obtained by fitting the respective experimental data to the denaturation[a], isodesmic[b] and nucleation-elongation[c] model.

Table S1. Thermodynamic parameters of supramolecular polymerization of **A** and **B**.[a]

c / µM	**A** ΔG_{298} / kJ mol^{-1}	**B** ΔG_{298} / kJ mol^{-1}
4	-47.6	-50.3
8	-44.0	-48.3
16	-41.7	-48.7
∅	-44.4	-49.1

Table S2. Thermodynamic parameters of supramolecular polymerization of **A** (THF/water (12/88)).[b]

c / µM	*c* / µM	T_m / K	Δs_{298} / kJ mol^{-1} T^{-1}	ΔH_{298} / kJ mol^{-1}	K	ΔG_{298} / kJ mol^{-1}
UV	2.5	303.7	-0.37	-109.8	506428.5	-32.6
Emission	2.5	300.8	-0.21	-94.8	433938.9	-32.2

Table S3. Thermodynamic parameters of supramolecular polymerization of **B** (THF/water (12/88)).[c]

c / µM	*c* / µM	ΔH_0 / kJ mol^{-1}	Δs_0 / kJ mol^{-1} T^{-1}	ΔH_{NP} / kJ mol^{-1}	T_e / K	ΔG_{298} / kJ mol^{-1}	K_{el}	K_{nucl}	σ
UV	2.5	-43.5	-0.02	-19.2	332.9	-43.5	400025.6	395.7	9.9E-4
Emission	2.5	-78.3	-0.05	-18.5	329.2	-52.0	400023.1	460.0	0.0012

Fluorescence Quantum Yields

The fluorescence quantum yields for **1**, **A** and **B** were calculated using Rhodamine 101 (MeOH) as standard (Φ_{ref} = 1.0) and using the following equation:

$$\Phi = \Phi_{ref} \frac{A_r\, I}{A\, I_f}$$

A: Absorption (set under 0.1) for reference and sample
I: Integral of emission-peak for reference and sample

Semiempirical calculations at the dispersion-corrected PM6 level in vacuum

Table S4. Heat of formation for Monomer (open and closed) and Tetramer structures of **B** (triethylene glycol gains have been replaced with methoxy chains).

	Heat of formation / kJ mol^{-1}
Monomer open	-920.4
Monomer closed	-935.3
Tetramer **B1**	-4131.1
Tetramer **B2**	-4138.1
Tetramer **B3**	-4154.5

Supplementary Figures

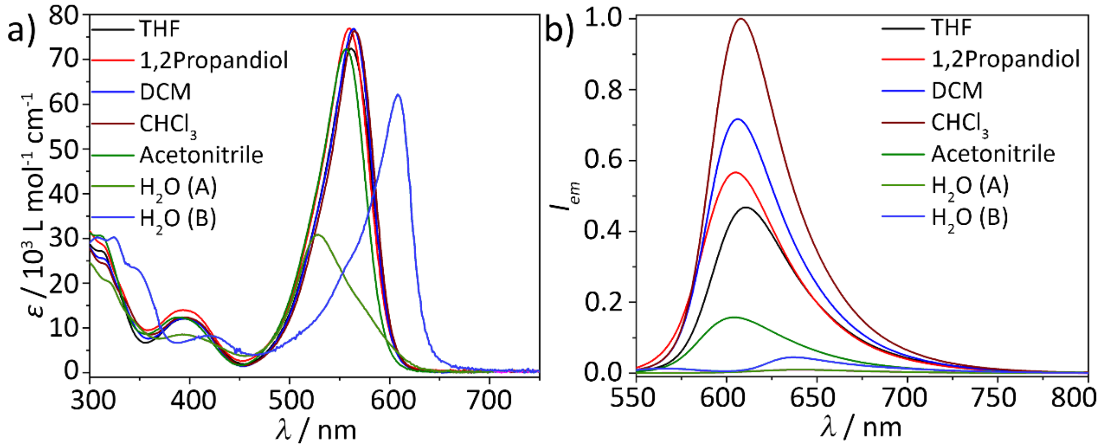

Figure S1. UV-Vis absorption (a) and emission spectra (b) of compound **1** in different solvents (c = 20 μM) at 298 K. For emission spectra, an excitation wavelength of λ = 530 nm was applied.

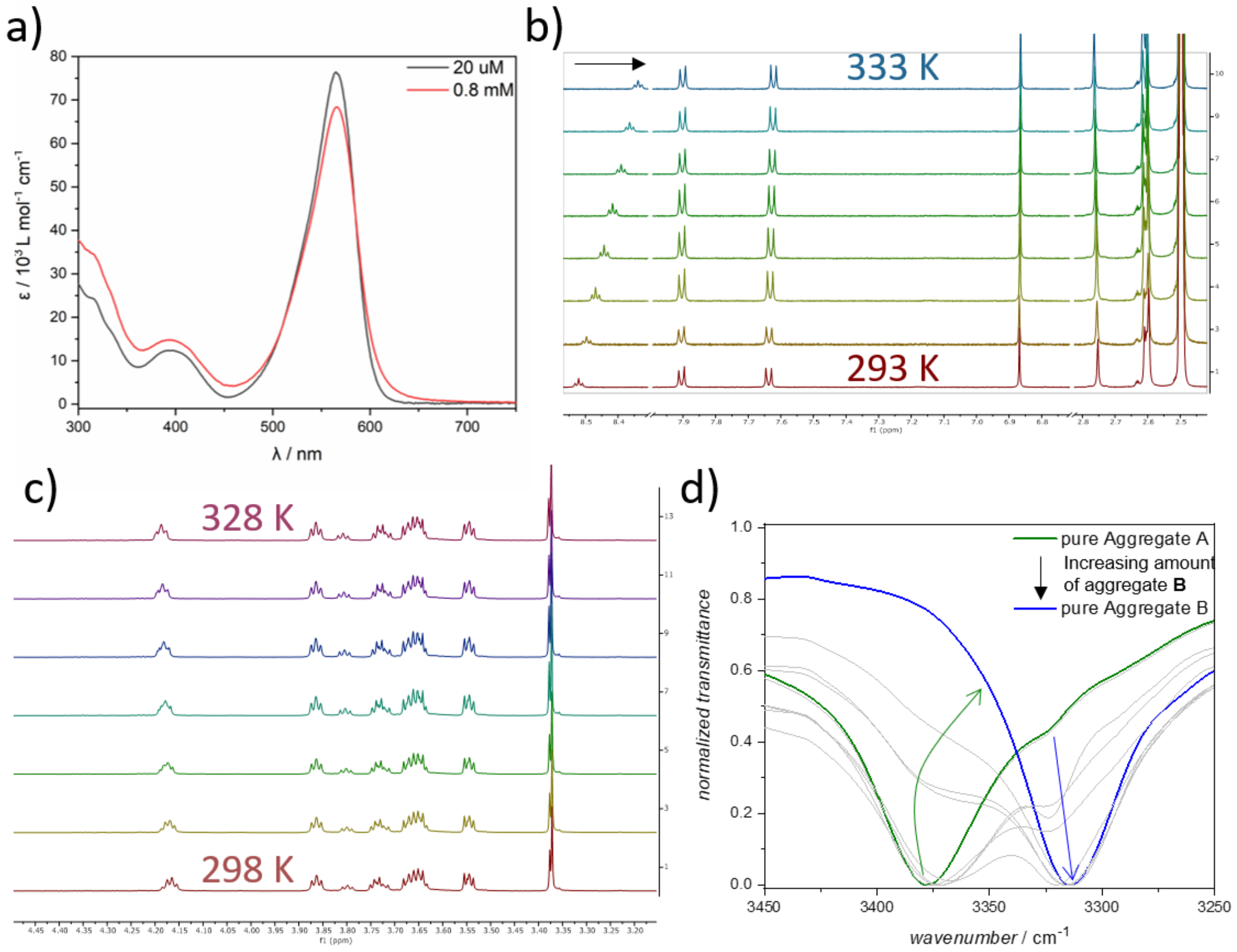

Figure S2. a) UV-Vis Spectrum of **1** in $CHCl_3$ at 20 μM and 0.8mM and RT showing similar monomer spectra. b) VT ^{1}H NMR of molecularly dissolved **1** in DMSO (1.0 mM, 293-333 K). c) VT ^{1}H NMR of molecularly dissolved **1** in $CHCl_3$ (0.8 mM, 298-328 K) showing the signals of the glycol chains. d) FTIR studies showing characteristic O-H/N-H stretching of mixtures of **A** and **B** (thin film from aqueous solution) at 298 K.

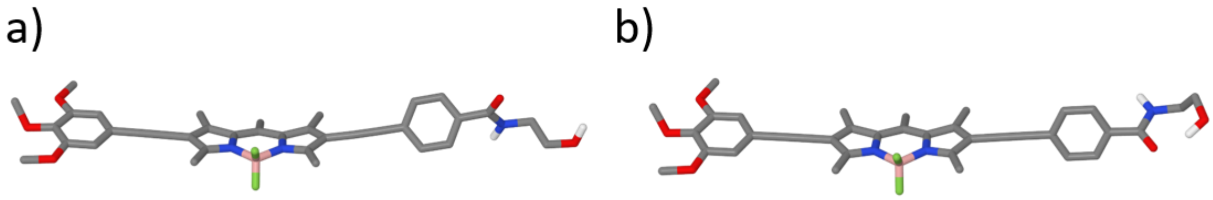

Figure S3. Open (a) and closed (b) monomer with methoxy chains optimized by semiempirical calculations at the dispersion-corrected PM6 level in vacuum

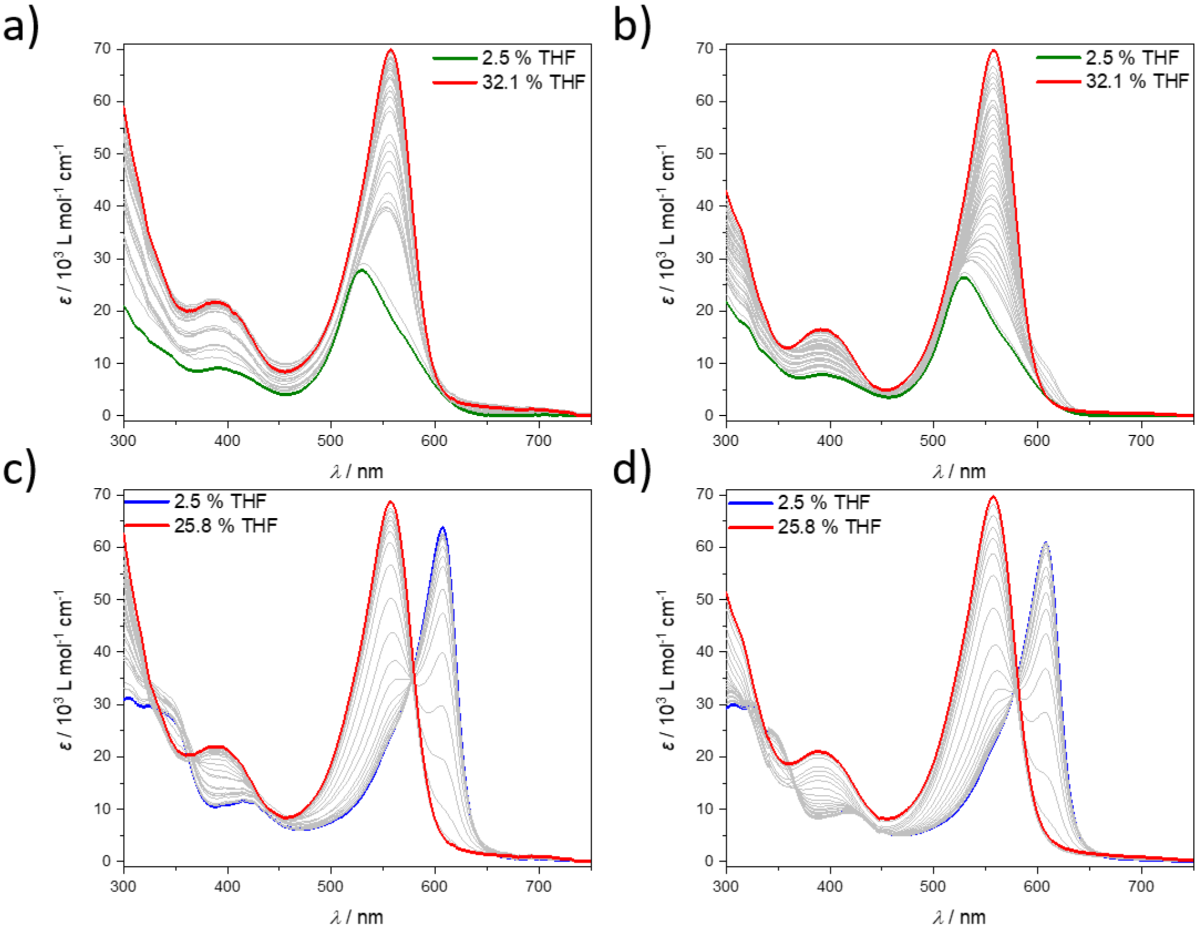

Figure S4. Denaturation UV-Vis Studies of **A** (4 μM (a) and 16 μM (b)) and **B** (b) (4 μM (c) and 16 μM (d)) at 298 K. For the studies, increasing amounts of **1** in THF are progressively injected to aqueous aggregates (with 2.5 vol% THF) **A** or **B** at the same concentration.

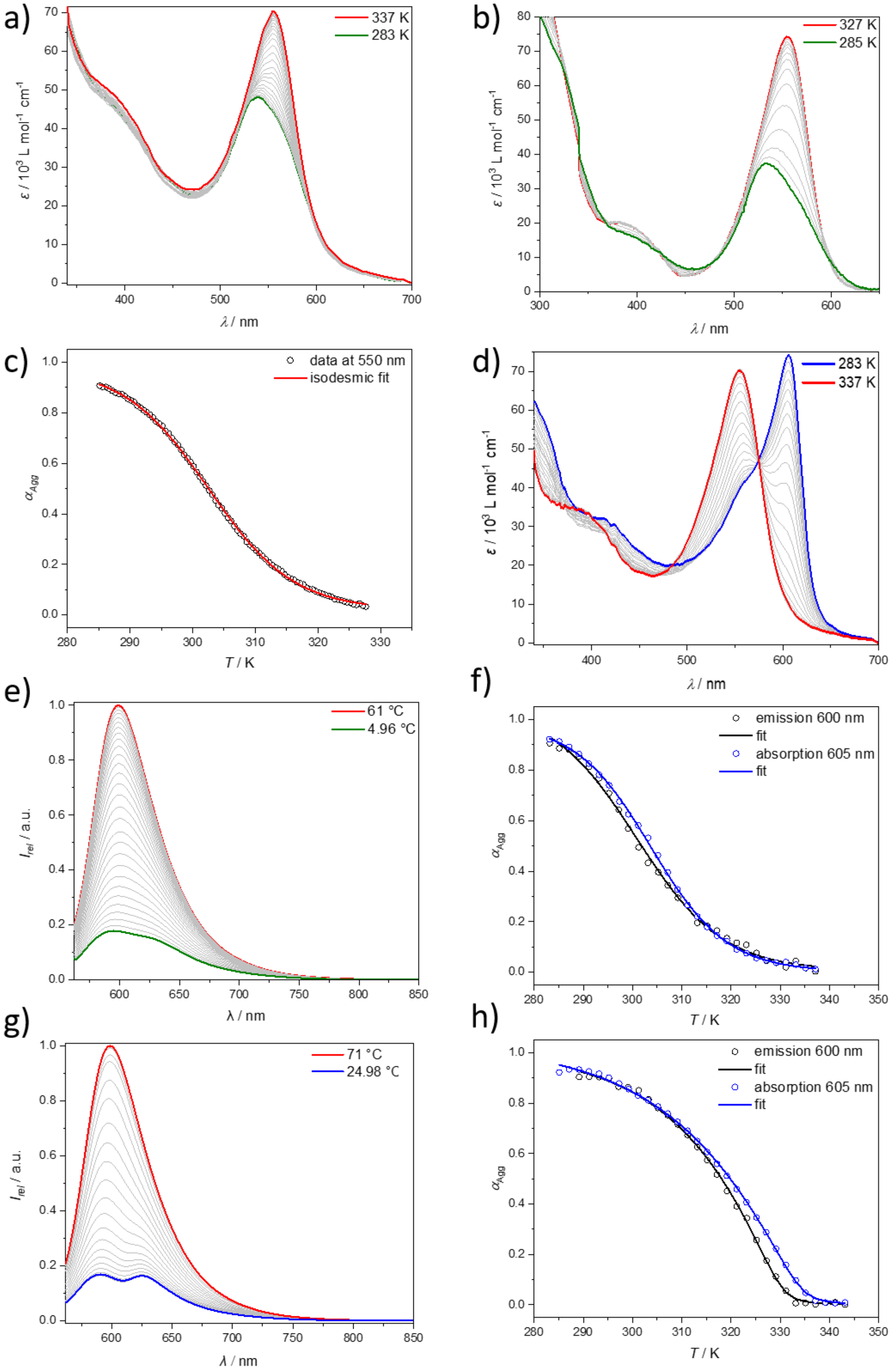

Figure S5. VT UV-Vis spectra of **A** (fast cooling: 2 Kmin^{-1} (a) and slow cooling: 0.1 Kmin^{-1} (b,c)) and **B** (heating: 2 Kmin^{-1} (d)) in water/THF (88/12) at c = 2.5 μM and fit to isodesmic model for Figure S4b (c). VT Emission spectra of **A** (e) and **B** (f) in water/THF (88/12) at c = 2.5 μM and using a cooling or respective heating rate of 2.0 K/min. Fit to nucleation elongation or isodesmic model for VT UV-Vis (a,d) and Emission studies (e,f) of **A** (g) and **B** (h).

We performed VT absorption and emission studies of **A** and **B** (Figure S5). Due to the high stability of aggregate **A** and **B** in aqueous solutions, even at high temperature and very dilute conditions (2.5 μM), the addition of 12 vol% THF is required to reach the monomer state.

Cooling a water/THF (88/12) solution at 2.5 μM from 340 K to 273 K induces the formation of the characteristic absorption spectrum of aggregate **A** (Figure S5a-b). This process follows the isodesmic mechanism, in the same manner as also observed in denaturation studies (Figure 3 and S4). VT emission studies using the same conditions exhibit the quenching of the monomer emission band upon cooling with the simultaneous rise of new aggregate bands at around 593 nm and 630 nm (Figure S5e). The thermodynamic analysis of these parameters calculated from these experiments yields a higher free Gibbs energy (-32.4 $kJmol^{-1}$) than the values obtained from denaturation studies (Figure 3, Table S2). This is justified considering the use of 12 vol% THF in VT studies, which is lowering the aggregation tendency.

On the other hand, for the mechanistic investigations of **B**, heating experiments were employed, as cooling of the monomer solution preferentially forms aggregate **A**, even at very low cooling rates of 0.1 $Kmin^{-1}$ (Figure S5a-b). Because of this retarded formation of **B** in comparison to **A**, heating ramps of already transformed **B** had to be used to monitor the full disassembly process. For VT UV-Vis, the same spectral transition as in denaturation studies is observed (Figure S5). The VT emission of **B** exhibits the disappearance of the two aggregate bands at 580 nm and 630 nm and the emergence of the monomer band upon heating (Figure S5g). Analysis of both experiments and fitting to the nucleation elongation model yielded thermodynamic parameters that are indeed also lower than denaturation studies (-43.5 $kJmol^{-1}$). However, comparison to the VT studies of **A**, which were performed with the same THF content, supports the higher stability of aggregate **B** (Figure S5h, Table S3).

The high nucleation penalty of **B**, which needs to be overcome during heating, is also reflected in the different values of elongation temperatures (ΔT_e = 4K) obtained using different spectral methods. Emission studies exhibit increased T_e values in comparison to absorption studies, as the former method is far more sensitive and is able to monitor this nucleation process (Figure S5h).

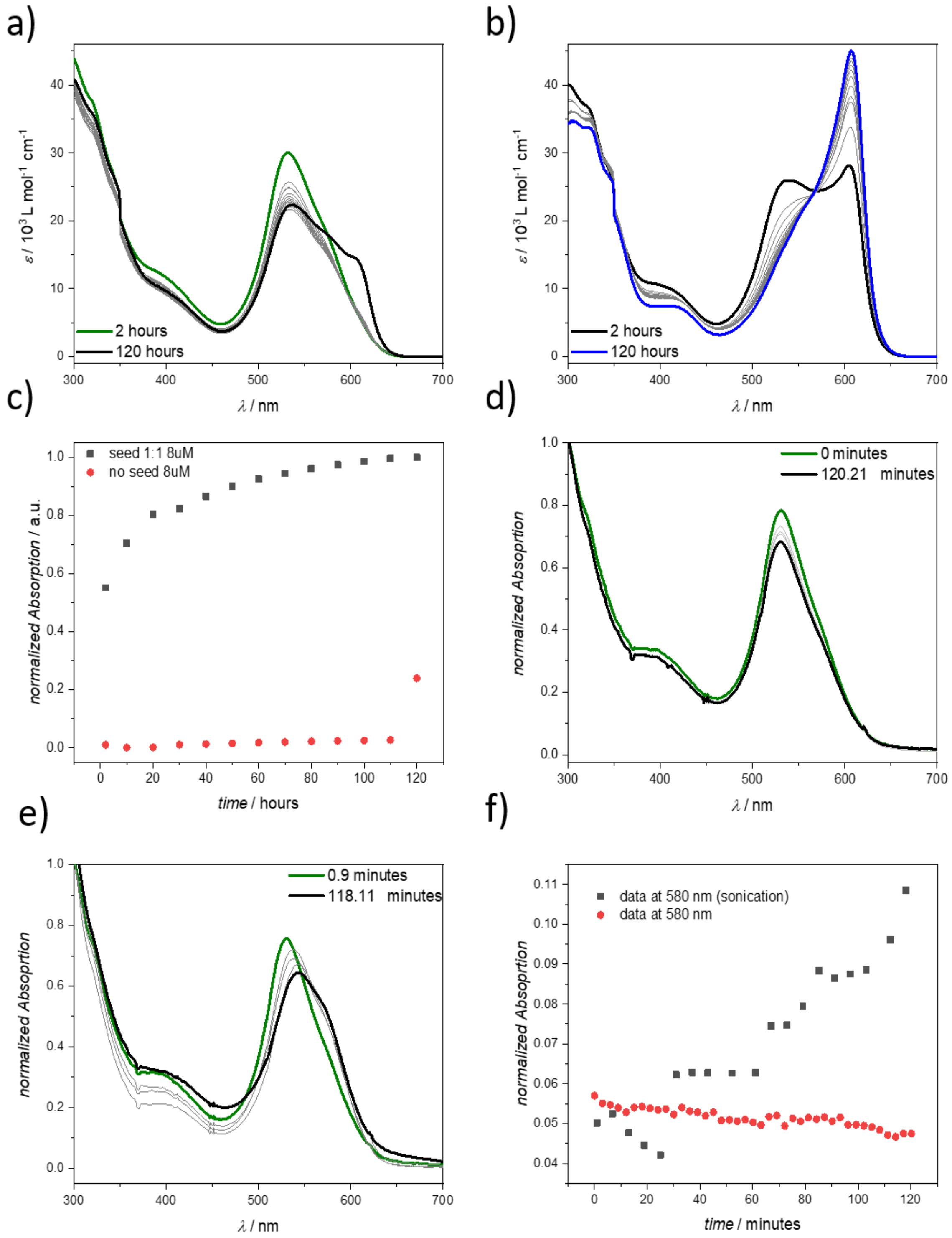

Figure S6. Time-dependent UV-vis studies monitoring the **A**→**B** transformation (1 vol% THF, c = 8 μM, 298 K): a) no seed b) **A** seeded with one equivalent **B**. c) comparison between seeding and without seeding. d) without sonication. e) with sonication. f) comparison between sonication and without sonication.

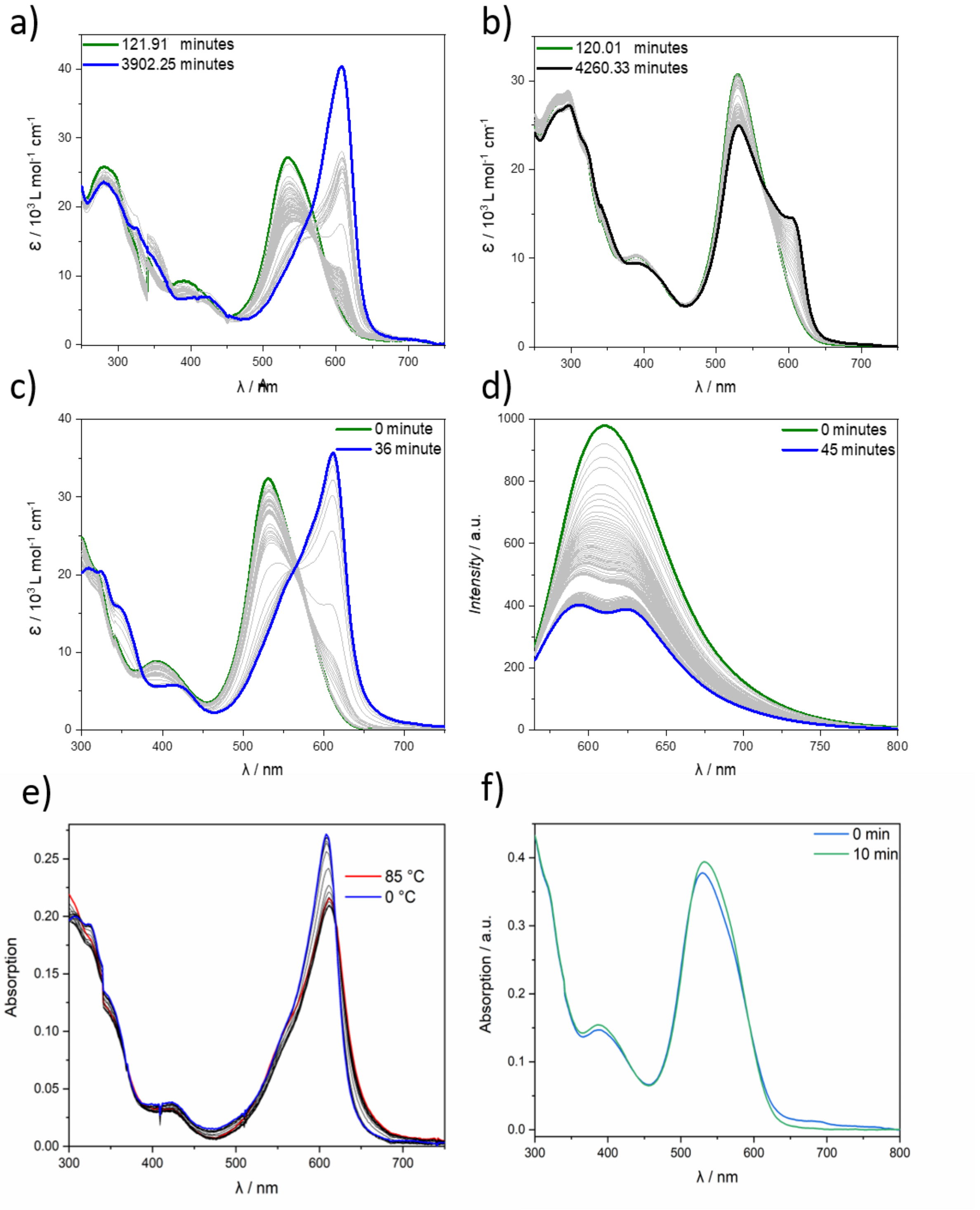

Figure S7. Time-dependent UV-vis studies monitoring the **A**→**B** transformation at multiple concentrations and temperatures with 10 vol% THF: a) 5 µM, 298 K, b) 20 µM, 298 K, c) 20 µM, 313 K without stirring. d) Time-dependent Fluorescence studies of **A** to **B** with 10 vol% THF at 20 µM and 313 K. e) Temperature-dependent UV-Vis studies of **B** in water with 10 vol% THF at 10 µM and heating rate of 2 K/min. f) UV-Vis spectra after solvophobic quenching of a hot THF Solution of **M** (0.2 mL, 60 °C) with water (1.8 mL) to yield **A** in a final concentration of 2 x 10^{-5} M (10 vol% THF, 60 °C)

After finding the appropriate experimental conditions (10 vol% THF, 25 °C or 40 °C), the kinetics of this transformation were monitored at different concentrations using UV-Vis and emission spectroscopy (Figure S7). For these studies, we ensured that the selected temperatures always lie below the elongation temperature using 10 vol% of THF in water (Figure S7e).

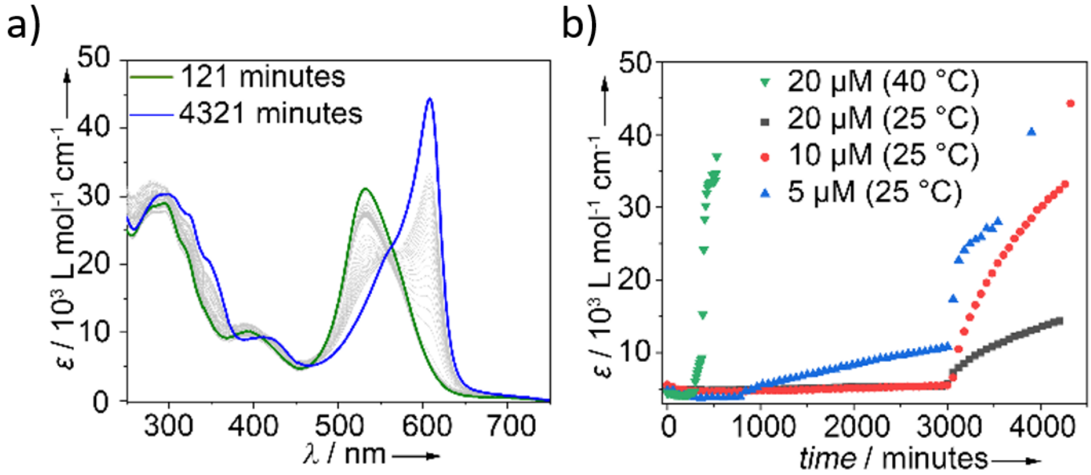

Figure S8. a) Time-dependent UV-Vis studies monitoring the **A**→**B** transformation in water/THF (9/1) (c = 10 µM) at 25 °C without stirring. b) Plot of molar extinction coefficient *vs.* time at λ = 610 nm at different concentrations.

Aggregate **B** is prone to form larger structures, which can be broken by roughly shaking, sonicating or stirring the solution, leading to more active polymer ends (faster transformation times, Figure S6-7). Another reasonable explanation is the sedimentation of these large structures. This is also apparent in Figure S8b, where at around 3000 minutes a sudden increase of the molar extinction coefficient (ε) is induced by roughly shaking the cuvettes.

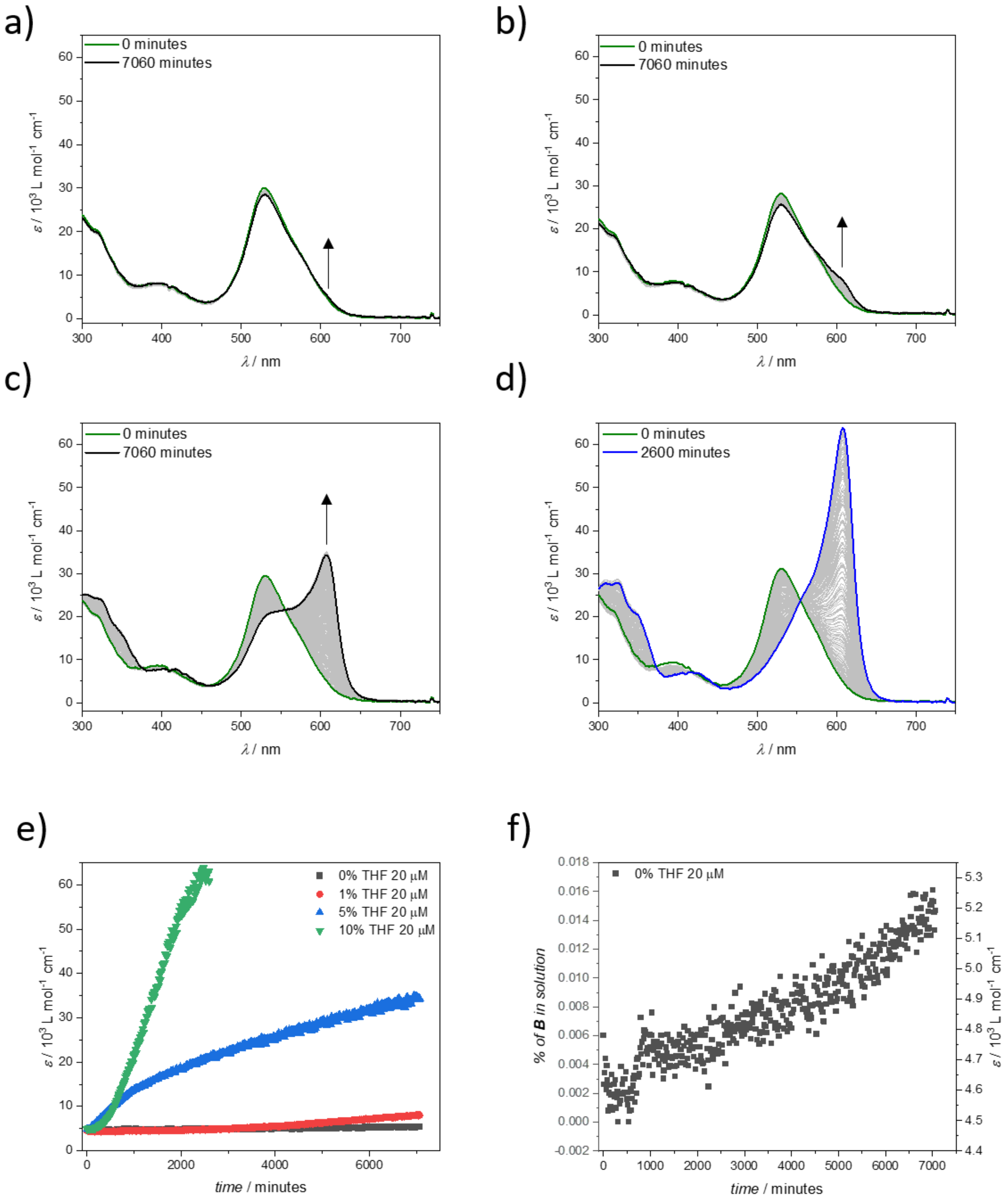

Figure S9. a) Time-dependent UV-vis studies monitoring the **A**→**B** transformation at multiple THF percentages (20 μM, 1000 rpm, 298 K): a) 0 vol% b) 1 vol% c) 5 vol% d) 10 vol%, e) Plot of molar extinction coefficient *vs.* time at λ = 610 nm, f) Plot of molar extinction coefficient and/or percentages of **B** in solution *vs.* time at λ = 610 nm and 0 vol% THF.

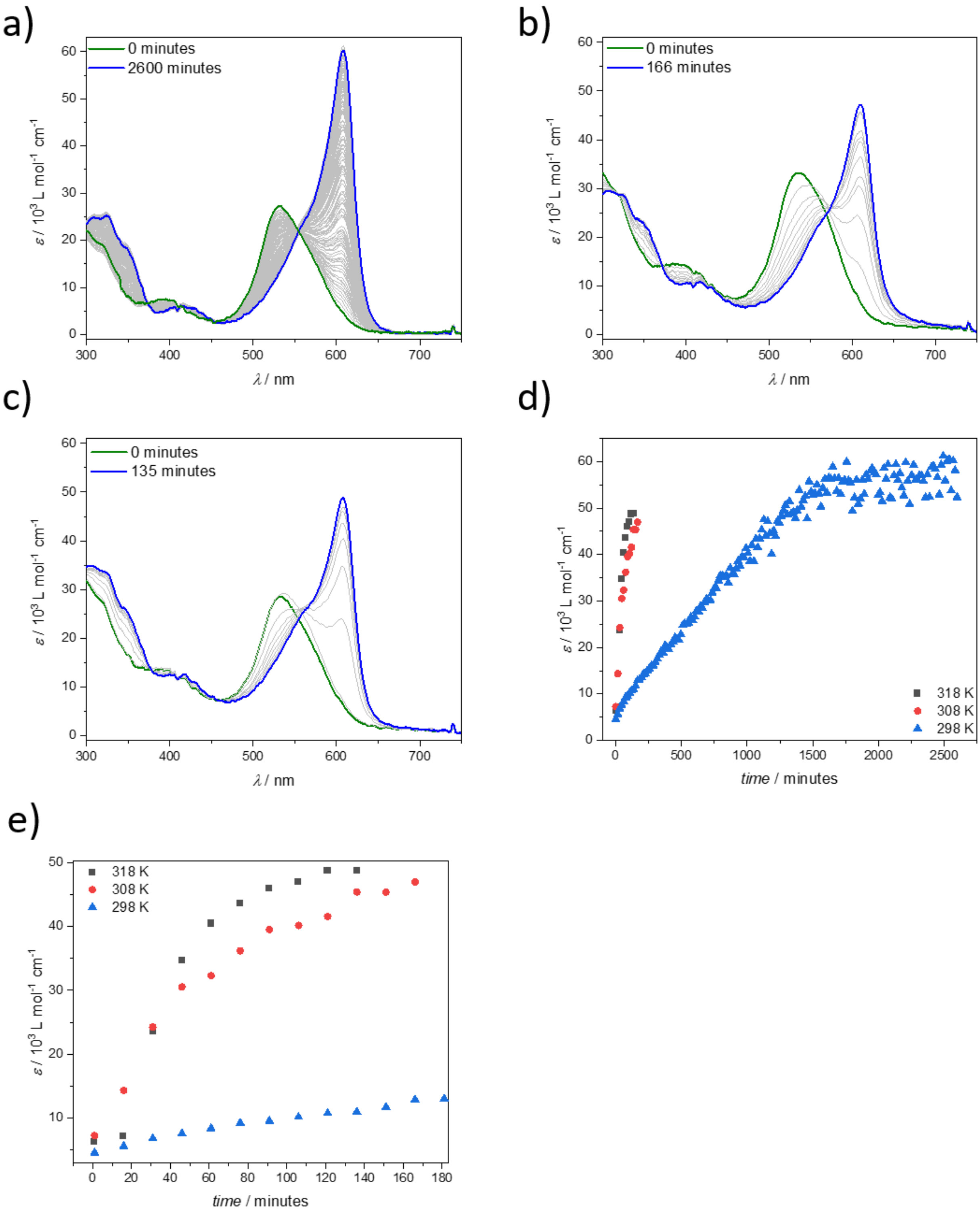

Figure S10. a) Time-dependent UV-vis studies monitoring the **A**→**B** transformation at multiple temperatures (10 μM, 10 vol% THF, 1000 rpm): a) 298 K, b) 305 K, c) 315 K, d) Plot of molar extinction coefficient *vs.* time at λ = 610 nm. e) enlarged region of the plot shown in d).

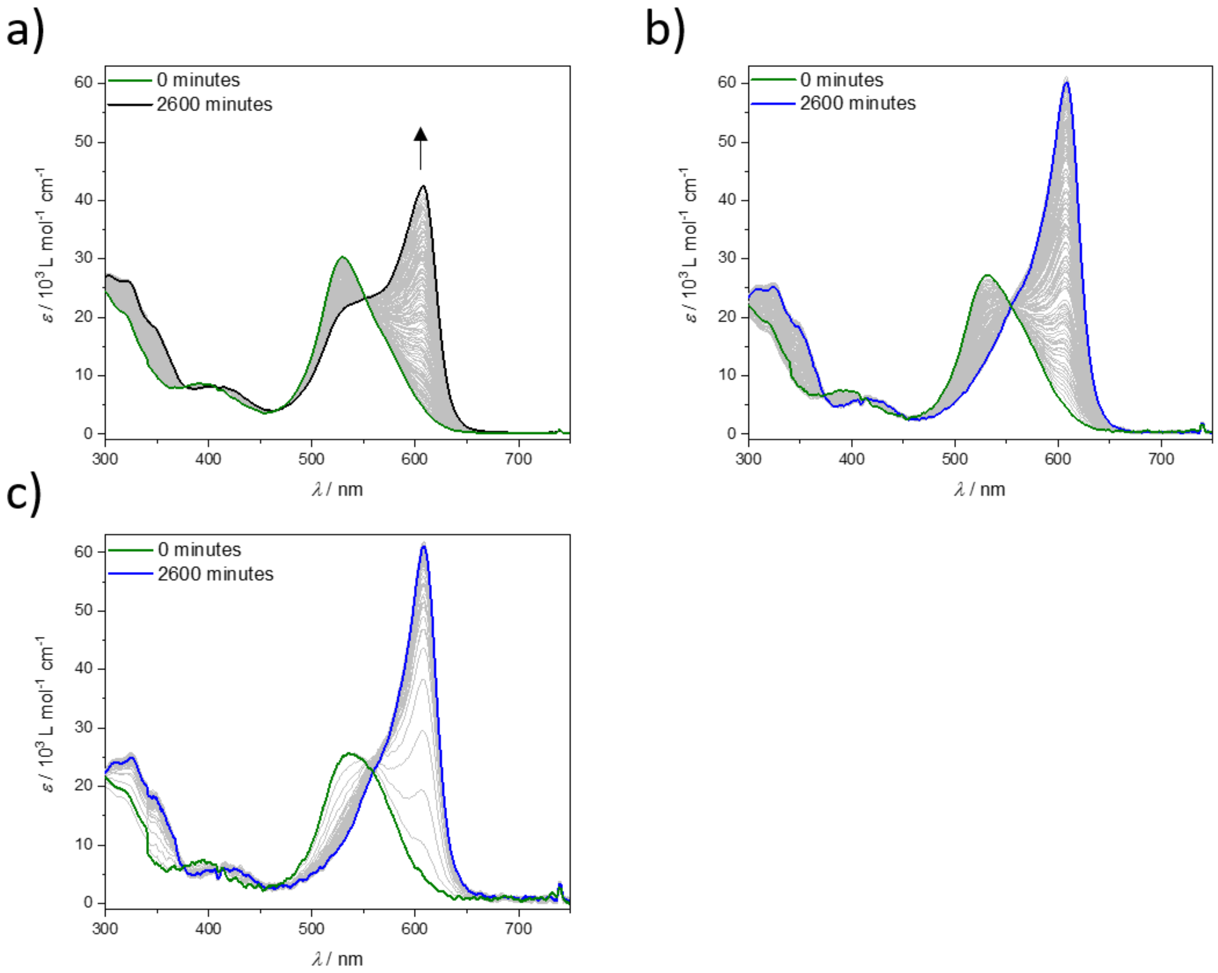

Figure S11. a) Time-dependent UV-vis studies monitoring the **A→B** transformation at multiple concentrations (10 vol% THF in water, 1000 rpm, 298 K): a) 40 μM b) 10 μM c) 5 μM.

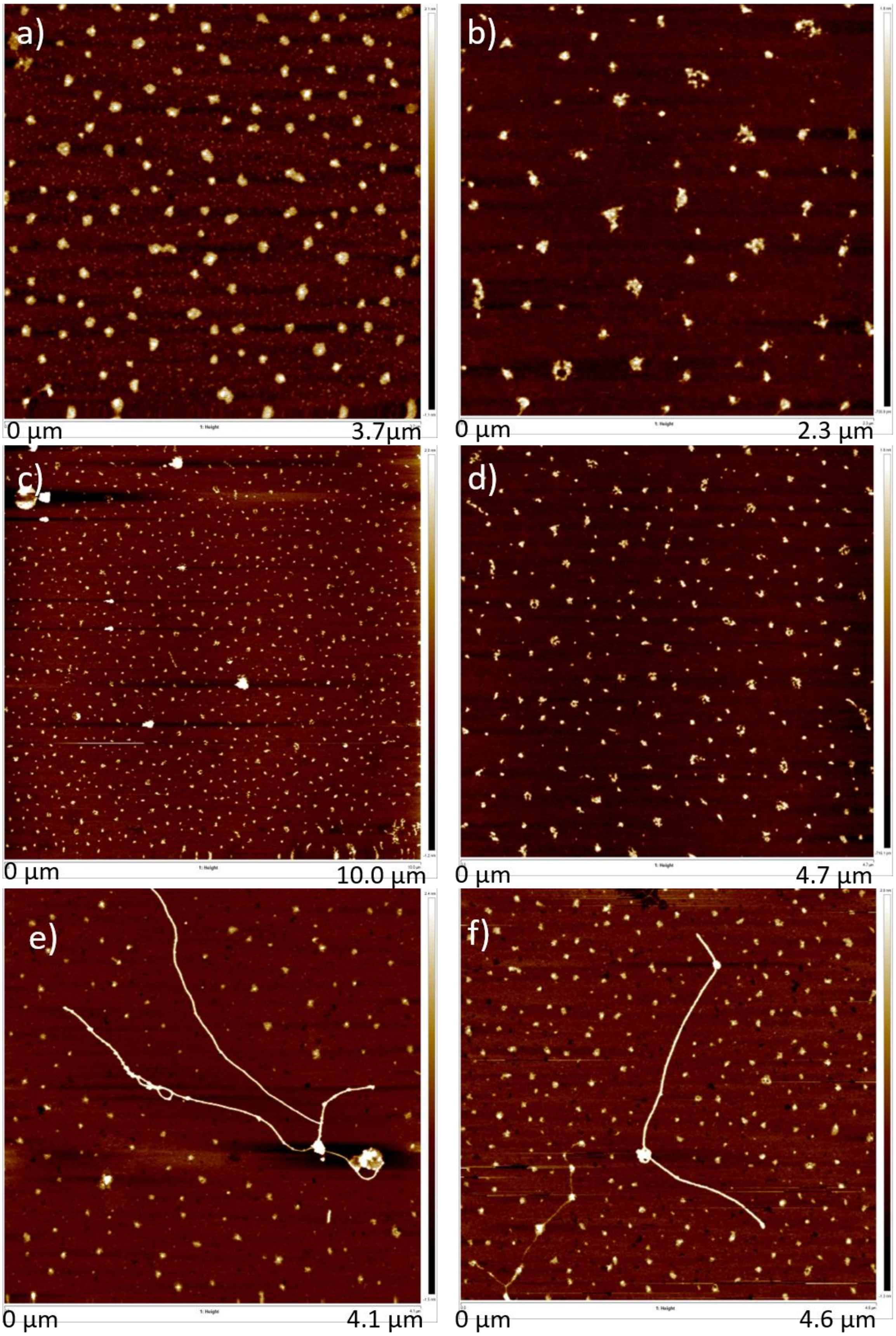

Figure S12. Time-dependent AFM-studies (8 µM, 1 vol% THF, aged at 298 K): a-d) 0 days, e-f) 7 days.

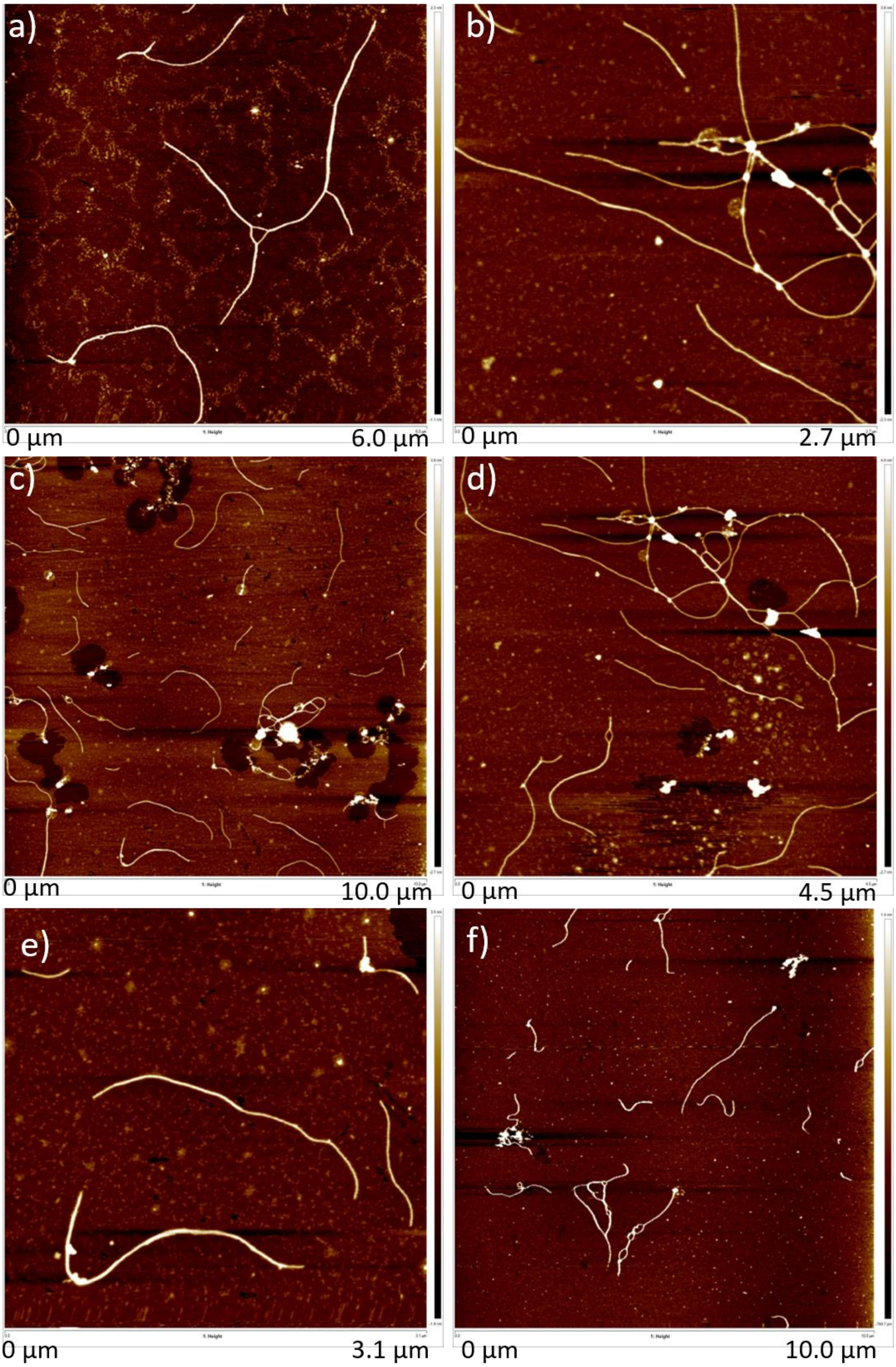

Figure S13. Time-dependent AFM-studies (8 µM, 1 vol% THF, aged at 298 K): a-f) 21 days.

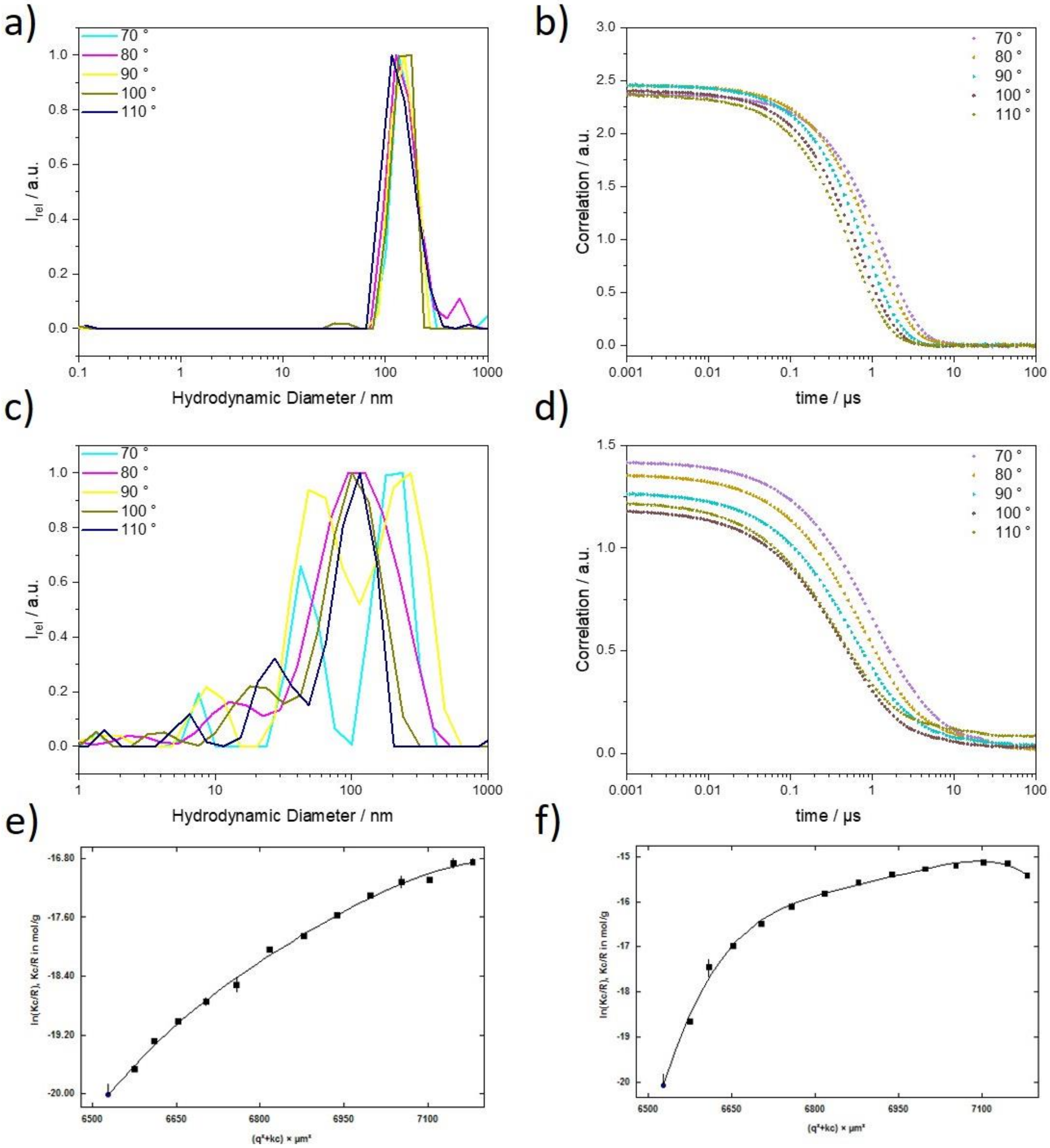

Figure S14. Angular-dependent DLS studies. Size distribution and corresponding correlation function for **A** (a,b) and **B** (c,d). SLS studies of aggregated species **A** (e) and **B** (f) and corresponding Guinier Plot. All studies have been performed at 298 K with the corresponding aggregate solutions (20 µM, 1 vol% THF).

The average hydrodynamic diameters of kinetic (**A**) and thermodynamic (**B**) species (D_H = 142 (**A**), 165 nm (**B**), obtained by CONTIN-Analysis of experimental DLS studies) have been related to the diameter of gyration (D_g = 167 (**A**), 341 nm (**B**), obtained by SLS studies and corresponding fit to the Guinier Plot) to unravel the differences in morphology. Note that 1 vol% THF has been used for DLS studies and soft phase of THF may also lead to an increased scattering signal, which can influence the results.

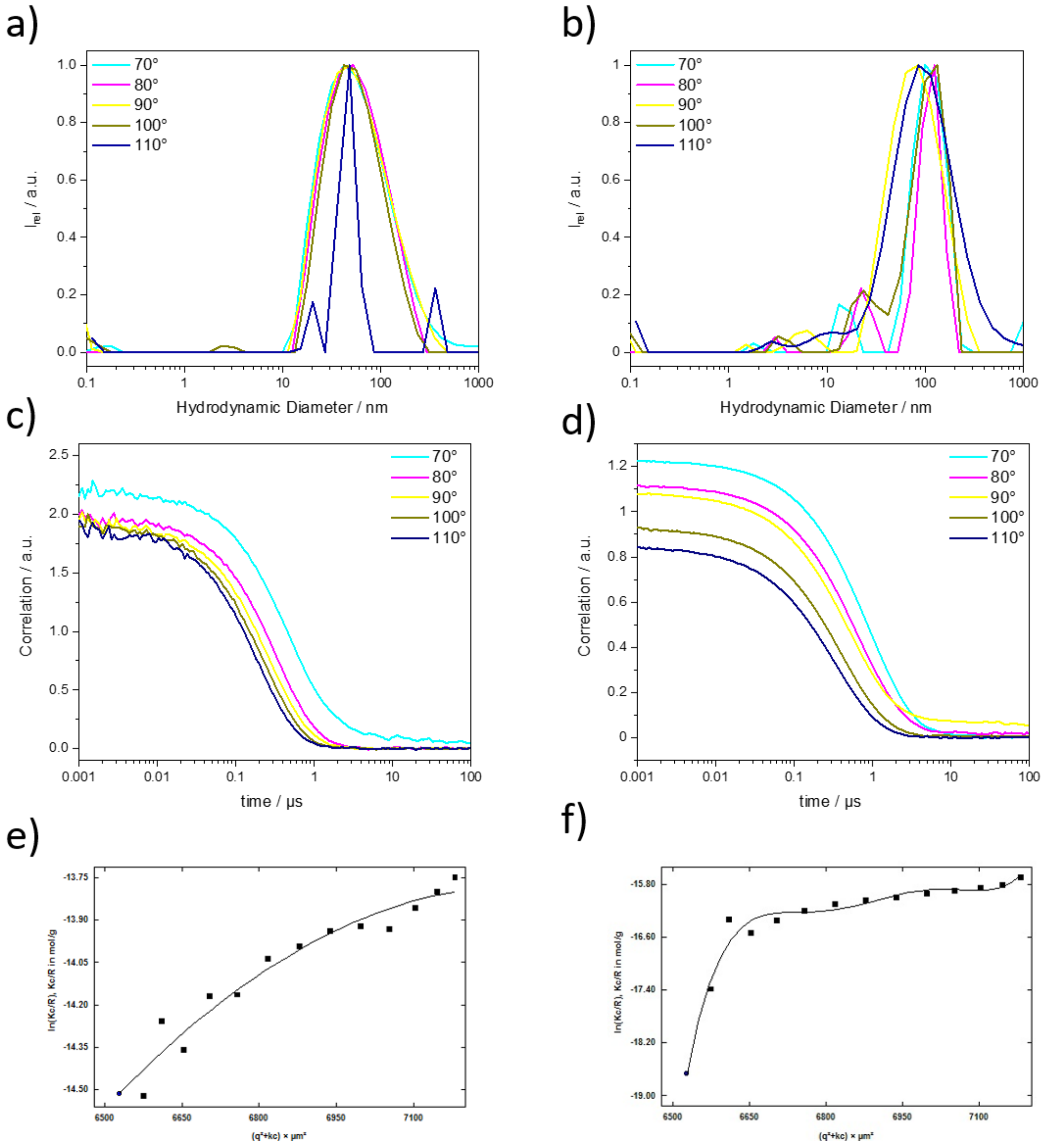

Figure S15. Angular-dependent DLS studies. Size distribution (a,b) and corresponding correlation function (c,d) for **A** (left) and **B** (right). SLS studies of aggregated species **A** (e) and **B** (f) and corresponding Guinier Plot. All studies have been performed at 298 K with the corresponding aggregate solutions (20 µM, ~0 vol% THF, ~100 vol% water).

The average hydrodynamic diameters of kinetic (**A**) and thermodynamic (**B**) species (D_H = 46 (**A**), 100 nm (**B**), obtained by CONTIN-Analysis of experimental DLS studies) have been related to the diameter of gyration (D_g = 74 (**A**), 353 nm (**B**), obtained by SLS studies and corresponding fit to the Guinier Plot) to unravel the differences in morphology. For ideal hard spheres D_g is 0.77 times the D_H value. However, for the kinetic species (**A**), the differences in comparison to this ideal system (D_g = 1.61 D_H) support the discoidal morphology, as found by AFM studies. For the thermodynamic species **B**, even larger differences between both values are achieved (D_g = 3.53 D_H), which points to the transformation to more anisotropic 1D structures, which is also in agreement with the AFM studies.

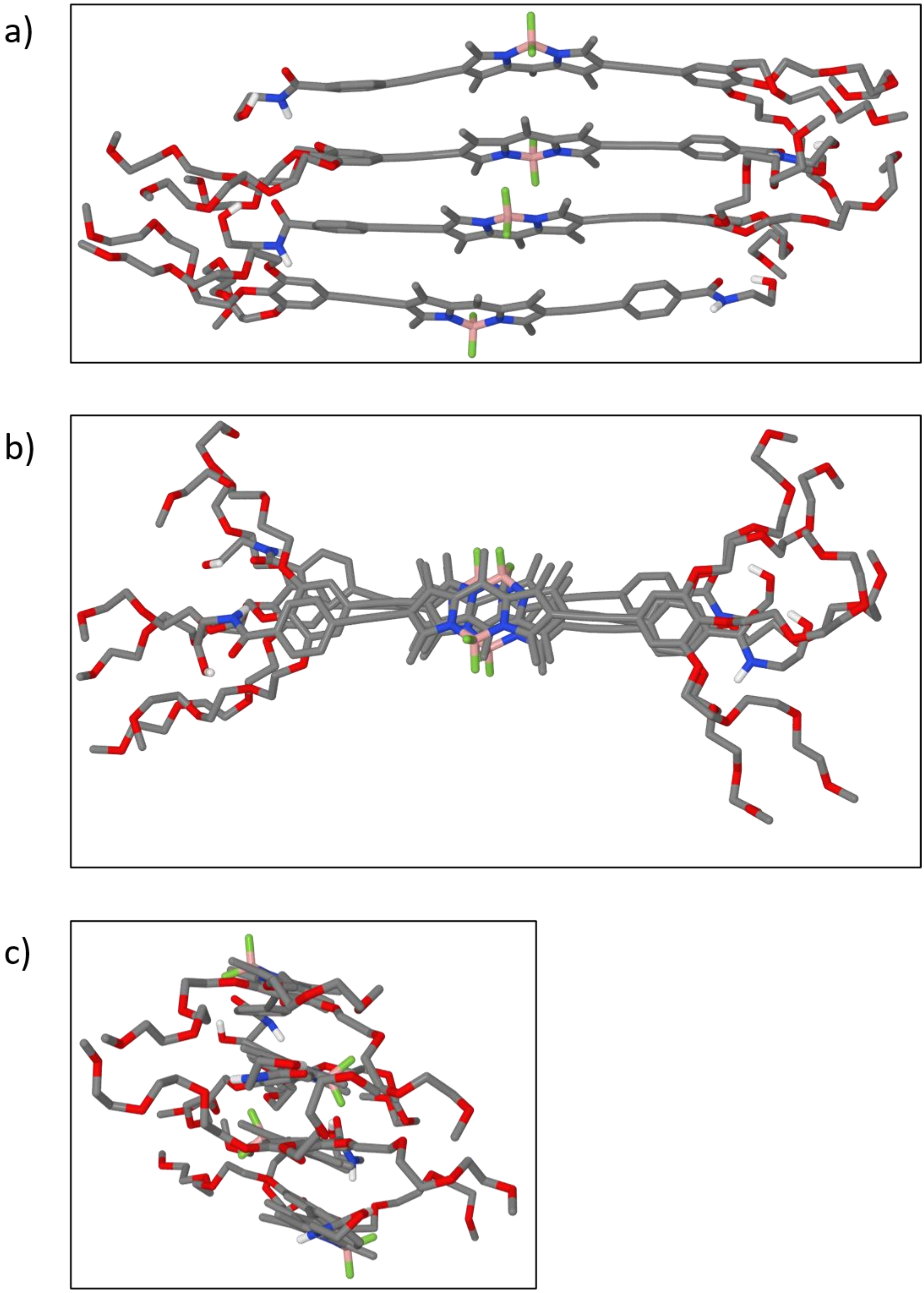

Figure S16. Tetramer structure of **A** optimized by semiempirical calculations at the dispersion-corrected PM6 level in vacuum.

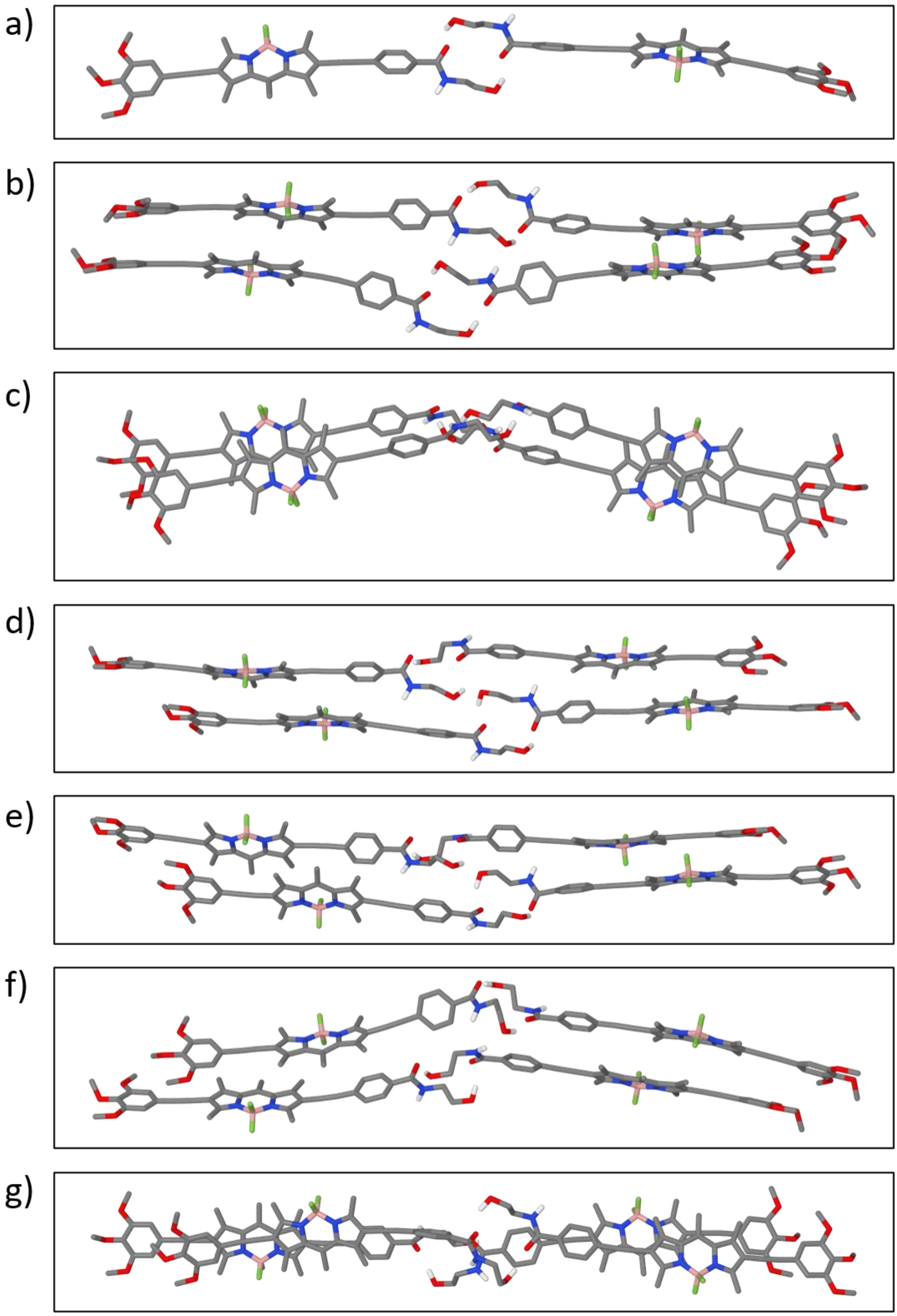

Figure S17. Dimer structure of **B** (a) and Tetramers for **B1** (b, c), **B2** (d, e) and **B3** (f, g) with methoxy chains optimized by semiempirical calculations at the dispersion-corrected PM6 level in vacuum.

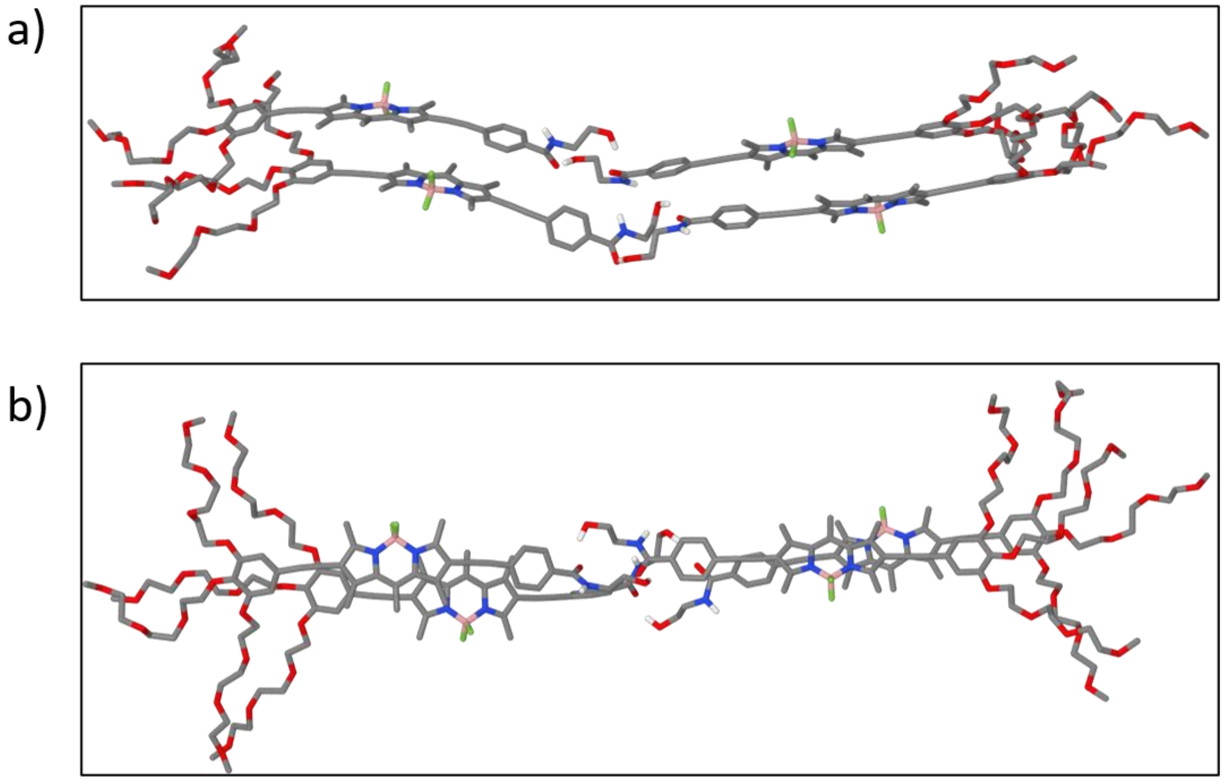

Figure S18. Tetramer structure of **B** optimized by semiempirical calculations at the dispersion-corrected PM6 level in vacuum.

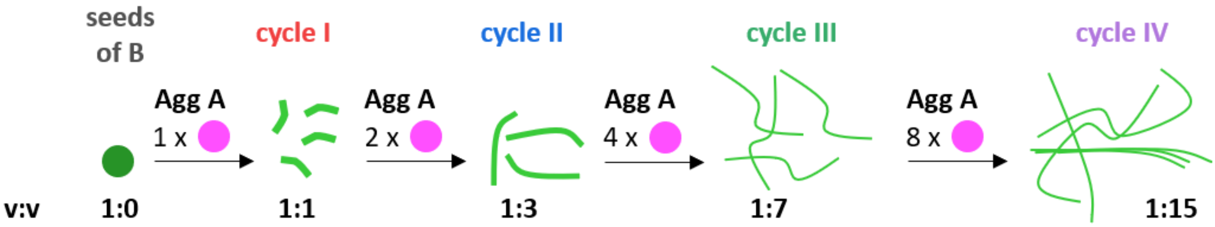

Figure S19. Experimental scheme for living supramolecular studies (1 vol% THF, c = 8 μM, 298 K).

J-type seeds were prepared by continuous sonication for 10 minutes. In contrast to the fibrous network of **Agg B**, the seeds of **B** exhibit a discoidal shape (Fig S21a-b). After 52 minutes, no changes in absorption for the J-seeds is detected (Figure S20a), and the living supramolecular polymerization was initiated by injection of the kinetically controlled aggregate **A** in a ratio of 1/1 (v/v). By plotting the maximum of aggregate **A** (530 nm) *vs.* time, the completion of the consumption of the monomer reservoir was observed (Figure 6). The elongation of **Agg B** at the expense of **Agg A** is finished after 31 minutes leading to small rods with an average size of 111 ± 24 nm. For the next cycles, the repeated addition of **Agg A** was performed at the same 1/1 (v/v) ratio. In this manner, the active ends (**Seeds B**) are diluted every cycle (**cycle 0**: 1:0, **cycle I**: 1:1, **cycle II**: 1:3, **cycle III**: 1:7, **cycle IV**: 1:15 (**B**-seeds/used kinetic **Agg A**), which in turn leads to a deceleration of the polymerization rate (Figure 6, S10) and further controlled continued elongation of **Agg B** (Figure 6, S20-22).
The length of 25 fibers was extracted from Figure S21-22 to calculate the average size obtained each cycle:

Table S5. Fiber length observed after each cycle of the living supramolecular polymerization experiments (1 vol% THF, c = 8 μM, 298 K).

	Fiber length of Cycle Í / µm	*Fiber length of Cycle ÍI / µm*	*Fiber length of Cycle ÍII / µm*	*Fiber length of Cycle ÍV / µm*
1	0.104	0.192	0.400	1.863
2	0.168	0.168	0.423	1.318
3	0.112	0.143	0.254	1.588
4	0.081	0.330	0.270	1.316
5	0.124	0.334	0.327	1.869
6	0.116	0.422	0.353	1.820
7	0.099	0.205	0.453	1.886
8	0.121	0.315	0.384	1.261
9	0.118	0.371	0.302	1.443
10	0.113	0.172	0.414	1.310
11	0.172	0.219	0.415	0.997
12	0.113	0.144	0.523	0.659
13	0.109	0.255	0.411	0.566
14	0.134	0.213	0.495	1.006
15	0.145	0.378	0.433	0.865
16	0.065	0.290	0.484	1.172
17	0.109	0.181	0.532	1.344
18	0.110	0.181	0.449	1.476
19	0.106	0.258	0.479	0.497
20	0.128	0.135	0.410	0.959
21	0.089	0.198	0.274	0.814
22	0.096	0.215	0.581	0.996
23	0.092	0.251	0.487	1.148
24	0.099	0.289	0.356	0.972
25	0.102	0.213	0.463	1.381
average	0.113	0.243	0.415	1.221
standard deviation	0.024	0.080	0.085	0.398
minimum	0.065	0.135	0.254	0.497
maximum	0.172	0.422	0.581	1.886

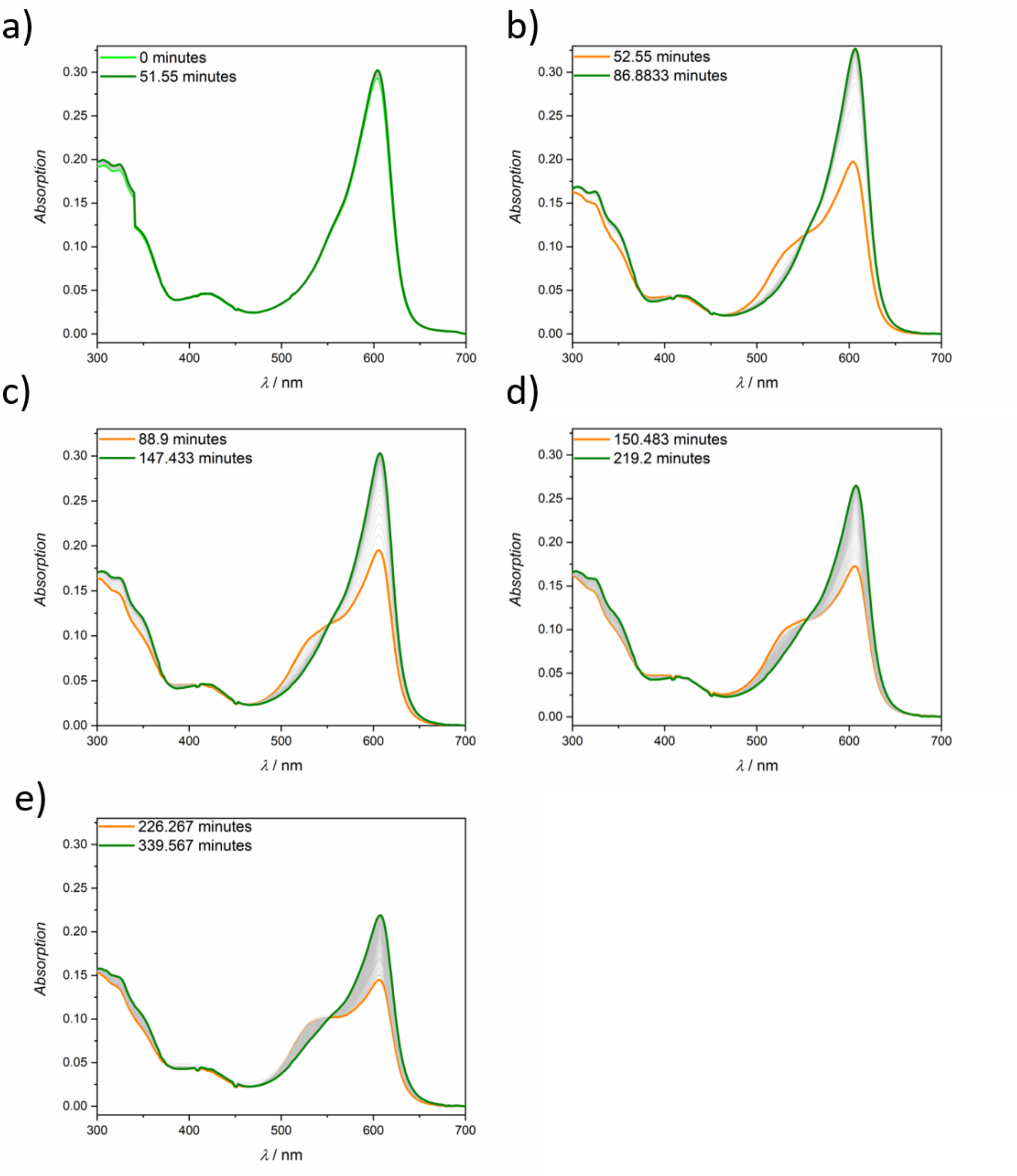

Figure S20. Living supramolecular UV-Vis absorption studies (1 vol% THF, c = 8 μM, 298 K): a) J-type seeds b) Cycle I: addition of aggregate **A** (1/1 (v/v), 1 vol% THF, c = 8 μM) to J-type seeds. c) Cycle II: addition of aggregate **A** (1/1 (v/v), 1 vol% THF, c = 8 μM) to elongated J-type fibers. d) Cycle III: addition of aggregate **A** (1/1 (v/v), 1 vol% THF, c = 8 μM) to elongated J-type fibers. e) Cycle IV: addition of aggregate **A** (1/1 (v/v), 1 vol% THF, c = 8 μM) to elongated J-type fibers.

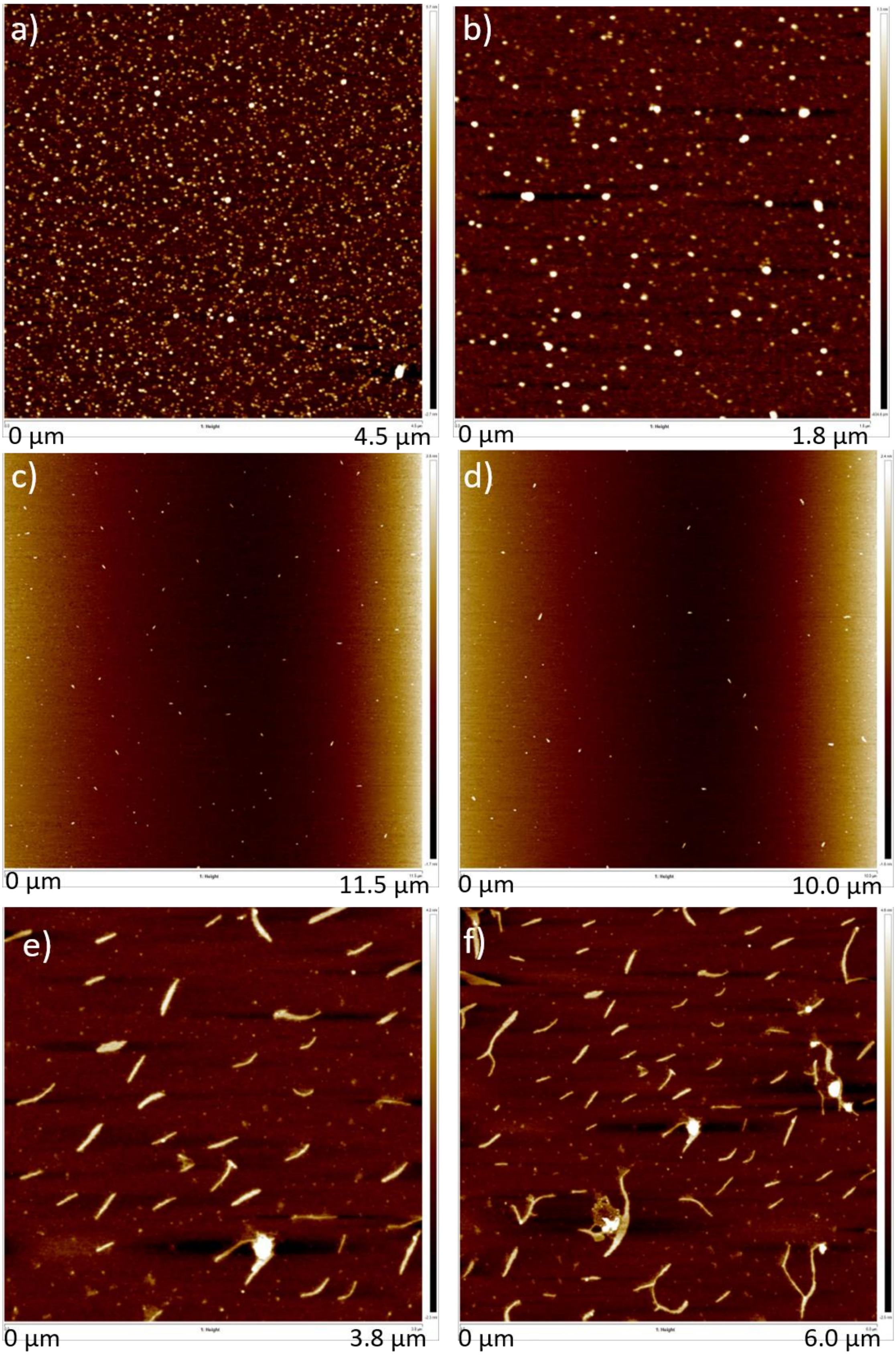

Figure S21. AFM studies (1 vol% THF, *c* = 8 µM, 298 K): a-b) J-type seeds c-d) Cycle I: addition of aggregate **A** (1/1 (v/v), 1 vol% THF, *c* = 8 µM) to J-type seeds. e-f) Cycle II: addition of aggregate **A** (1/1 (v/v), 1 vol% THF, *c* = 8 µM) to elongated J-type fibers.

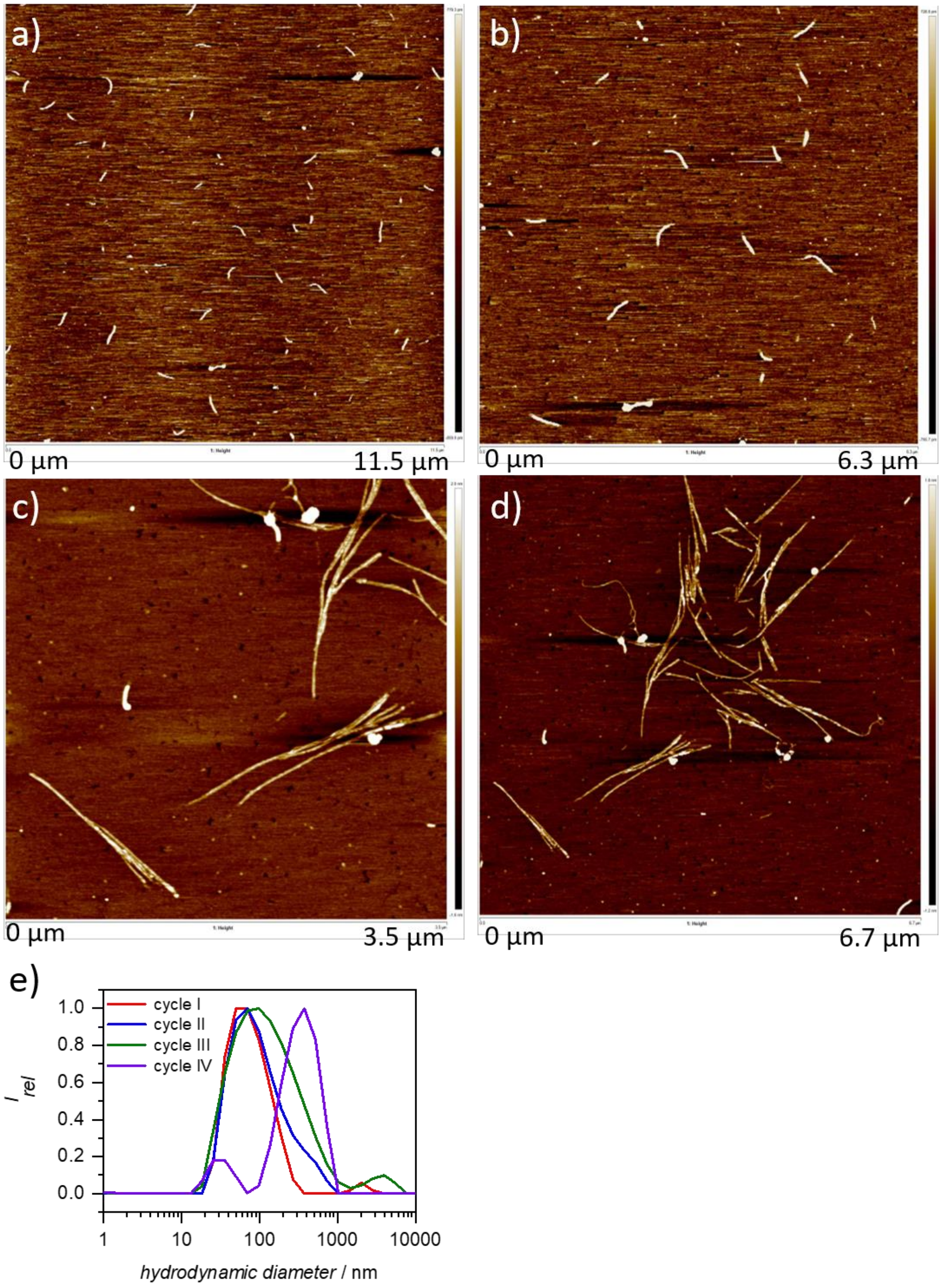

Figure S22. AFM studies (1 vol% THF, *c* = 8 µM, 298 K): a-b) Cycle III: addition of aggregate **A** (1/1 (v/v), 1 vol% THF, *c* = 8 µM) to elongated J-type fibres. c-d) Cycle IV: addition of aggregate **A** (1/1 (v/v), 1 vol% THF, *c* = 8 µM) to elongated J-type fibres. e) DLS size distribution of fibres after each cycle (scattering angle = 90°).

Note that 1 vol% THF has been used for DLS studies and soft phase of THF may also lead to an increased scattering signal, which can influence the results.
